# Lactate-induced H3K4me3 histone mark promotes PLOD1-driven prostate cancer invasion

**DOI:** 10.1038/s41419-026-09091-4

**Published:** 2026-07-07

**Authors:** Marta Iozzo, Luigi Ippolito, Erica Pranzini, Simone Romagnoli, Giulia Gangarossa, Caterina Grillo, Sara Roccabianca, Giada Sandrini, Matteo Benelli, Giuseppina Comito, Elisa Giannoni, Paola Chiarugi

**Affiliations:** 1 Department of Experimental and Clinical Biomedical Sciences, University of Florence, Florence, Italy; 2 Division of Theoretical Systems Biology, German Cancer Research Center (DKFZ), Heidelberg, Germany

**Keywords:** Cancer metabolism, Prostate cancer

## Abstract

Prostate cancer (PCa) progression is strongly influenced by the metabolites available in the tumor microenvironment (TME), including lactic acid (LA), which is actively imported by PCa cells to boost mitochondrial metabolism and drive de novo collagen synthesis, sustaining increased malignancy. LA exploitation promotes the unbalance of tricarboxylic acid (TCA) cycle intermediates, particularly succinate and fumarate, well-known epigenetic modifiers for histone (de)methylation. Here, we show that the LA-induced increase in succinate levels affects the activating H3K4me3 methylation mark in PCa cells, promoting a pro-invasive phenotype. Notably, pharmacological targeting of H3K4me3 using OICR-9429 reduces LA-enhanced PCa cell invasiveness. Moreover, LA-induced H3K4me3 enrichment regulates the expression of procollagen-Lysine,2-Oxoglutarate 5-Dioxygenase 1 (PLOD1), a key enzyme involved in collagen maturation. Genetic impairment of PLOD1 reduces the LA-driven invasive potential of PCa cells, thereby highlighting PLOD1 as a crucial epigenetically regulated mediator of tumor invasion. Overall, our findings uncover a novel LA-fuelled metabolic-epigenetic axis that promotes the H3K4me3-mediated PLOD1 upregulation, consequently fostering PCa aggressiveness and unveiling a potential therapeutic vulnerability.

## Introduction

Increasing evidence highlights a deep interplay between tumor metabolism and transcriptional regulation [1–4]. Several metabolites not only sustain biosynthetic and energetic demands but also serve as epigenetic regulators, modulating chromatin architecture and gene expression [5–8]. In cancer cells, this dynamic crosstalk facilitates flexible adaptation to challenging conditions during cancer progression. Among these, lactic acid (LA), acts as a central oncometabolite acting as a metabolic substrate and/or signalling molecule sustaining the development of different types of tumor [9–14]. In PCa, LA is abundantly secreted by cancer-associated fibroblasts (CAFs) and taken up by PCa cells, through monocarboxylate transporter 1 (MCT1), where it fuels oxidative metabolism, support invasive potential, and sustains pro-metastatic behaviour in vitro and in vivo [10]. The LA-induced mitochondrial rewiring drives an unbalance in the levels of key metabolic intermediates of TCA which can be exploited to sustain different functions such as lipid anabolism [10] and extracellular matrix (ECM) remodelling [15]. In addition, recent studies suggest that LA modulates histone acetylation in PCa cells, by exploiting citrate-mediated lipid droplets (LDs) formation and subsequent production of acetyl-CoA [10]. Similar mechanisms have been reported in macrophages, where LA-derived citrate is converted to acetyl-CoA via ATP-citrate lyase, fuelling histone acetylation and driving immunosuppressive M2 polarization [16]. Further sustaining its regulatory roles, LA also serves as a critical substrate for histone lactylation, a recently identified post-translational modification with a key role in tumor progression [7, 9].

Among epigenetic mechanisms, histone methylation plays a central role in shaping the transcriptional landscape that underlies malignant traits. Both activating - H3K4me3, H3K36me3 - and repressive - H3K9me3, H3K27me3 - methylation marks contribute to the establishment and maintenance of transcriptional states that support cancer cell adaptation and progression [17, 18]. However, the direct involvement of LA in modulating this transcriptional modification remains poorly understood.

Methylation of lysine residues, catalyzed by histone methyltransferases (HMTs), is one of the most common histone modifications [19, 20]. This modification is dynamic and reversible, under the influence of two main classes of histone demethylases: the FAD-dependent lysine-specific demethylase family and the α-ketoglutarate-dependent Jumonji C (JmjC) domain-containing family. Interestingly, TCA cycle intermediates such as succinate and fumarate can inhibit α-ketoglutarate (α-KG)-dependent dioxygenases, including the JmjC histone demethylases, thereby promoting a hypermethylated chromatin state that activates key oncogenic transcriptional programs (e.g., involved in cell proliferation, migration, and survival), ultimately driving tumorigenesis [21–25].

Here, we characterize a previously undescribed role of LA in modulating histone methylation. Specifically, we underscored that LA can remodel H3K4me3 profile in PCa cells, thereby reshaping the expression of genes regulating tumor cell invasiveness. Specifically, PLOD1 emerged as a key H3K4me3-regulated gene, which mediates PCa cell aggressiveness in LA-rich conditions.

## Results

### H3K4me3 is enriched in aggressive PCa in a LA-driven manner

**Fig. 1:**
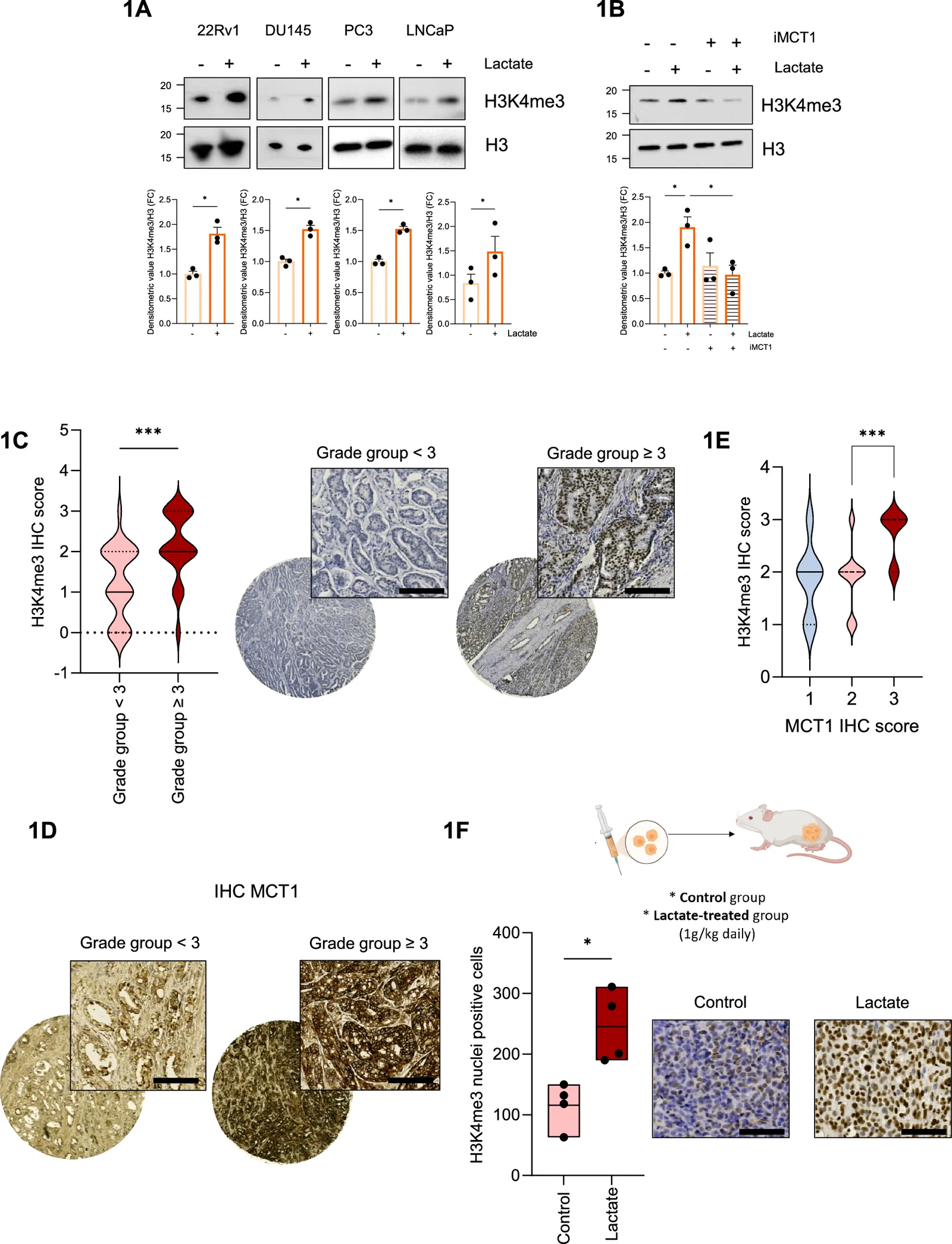
LA exposure increases H3K4me3 levels in PCa. **A** H3K4me3 levels evaluated by western blot (WB) analysis in 22Rv1, DU145, PC3 and LNCaP cells treated or not with LA (20 mM, 48 h). H3 was used as loading control. Densitometric analysis of H3K4me3/H3 ratio is shown (*n* = 3). **B** H3K4me3 levels evaluated by WB analysis in 22Rv1 cells treated with LA ± MCT1 inhibitor (AR-C155858, 40 μM; MCT1i, 48 h). H3 was used as a loading control. Densitometric analysis of H3K4me3/H3 ratio is shown (*n* = 3). **C** Quantification of H3K4me3 levels by IHC in human PCa TMA cores stratified by Grade Group (low-grade, Grade Group < 3; high-grade, Grade Group ≥ 3; *n* = 46). Representative images are shown (magnification 5x and 20x), scale bar: 200 μm. **D** MCT1 levels by IHC in human PCa TMA cores stratified by Grade Group (low-grade, Grade Group < 3; high-grade, Grade Group ≥ 3; *n* = 46). Representative images shown (magnification 5x and 20x), scale bar: 200 μm. (**E**) Correlation analysis of MCT1 and H3K4me3 in PCa TMA. Signal intensity was assessed by H-score (0-3) in matched cores stained for MCT1 or H3K4me3. **F** Ex vivo immunostaining of H3K4me3 in PCa xenograft tissues from mice treated or not with LA (*n* = 4). Representative images shown, scale bar: 20 μm. Created with BioRender.com (**F**). Data are represented as means ± SEM of *n* = 3 biological replicates unless otherwise stated. Statistical significance was assessed using two-tailed unpaired Student’s t-test (**C**, **E**) or Mann-Whitney test (**D**) (*P < 0.05; ***P < 0.001).

Given the unexplored role of LA in histone methylation regulation, we assessed both active and repressive histone methylation marks (i.e., H3K4me3, H3K36me3, and H3K9me3, H3K27me3, respectively) following treatment with 20 mM LA (a concentration reflecting the intratumoral levels observed in different tumor types, including PCa [15, 26]) in PCa cells. Notably, only H3K4me3 was significantly increased in the subjected cell lines upon LA treatment (Fig. 1A). In contrast, H3K36me3, H3K27me3, and H3K9me3 levels do not show relevant modulation (Fig. S1A).

Notably, pharmacological inhibition of LA uptake using AR-C155858, a selective inhibitor of MCT1 (iMCT1), resulted in a strong reduction in LA-induced H3K4me3 enrichment (Fig. 1B) in 22Rv1 cells, demonstrating a direct dependence on LA import.

To investigate whether the H3K4me3 correlates with the progressive increase in tumor grading in PCa, we performed an immunohistochemistry (IHC) analysis on a human PCa tissue microarray (TMA). H3K4me3 was significantly elevated in high Grade group PCa tumors compared to low-grade ones (Fig. 1C), indicating a clear correlation of H3K4me3 with aggressive patient-derived PCa tumors. Notably, this pattern correlates with the previously demonstrated increase in MCT1 expression (Fig. 1D) along with tumor grading in PCa [10]. In addition, H3K4me3 levels were markedly elevated in tumors exhibiting high MCT1 expression (Fig. 1E), supporting a putative role of H3K4me3 in LA-exploiting (i.e., MCT1-high) PCa tissues.

Noteworthy, LA exposure also promoted an enhanced invasive capacity in 22Rv1 cells (Fig. S1B). This invasive phenotype was attenuated by MCT1 inhibition, suggesting a functional link between LA uptake and 22Rv1 cell motility (Fig. S1C).

To further corroborate this finding, we used ex vivo tumor tissues derived from PCa cells xenografted in mice *i.p*. treated with LA [15]. IHC analysis showed a strong H3K4me3 staining in PCa xenografts experiencing LA treatment (Fig. 1F), further demonstrating that higher availability of this metabolite contributes to sustain this methylation mark.

To strengthen this observation, we analyzed H3K4me3 expression in a 3D in vitro stromalized PCa model [27], which mimics the complex interplay established at the primary tumor between PCa and stromal (e.g., cancer-associated fibroblasts, CAFs) cell populations. IHC analysis revealed elevated H3K4me3 levels in the presence of CAFs (Figure S1C), supporting the notion that stroma-derived lactate contributes to epigenetic remodelling in PCa. This model recapitulates key features of tumor-stroma interactions and underscores the relevance of the microenvironment in shaping the epigenetic landscape, thus validating our in vivo findings in a complementary ex vivo setting.

Collectively, these findings underline the existence of a correlation between high environmental LA and increased H3K4me3 mark which may contribute to enhanced aggressiveness in PCa.

### LA-dependent changes in succinate levels improve H3K4me3 and PCa cell invasion

LA has been previously associated with epigenetic alterations (i.e., histone acetylation) in PCa, due to increased levels of citrate fuelling fatty acid synthesis and LDs assembly, ultimately providing acetyl moieties [10]. However, other TCA cycle intermediates such as succinate and fumarate affect DNA/histone hypermethylation, by competing with α-KG and inhibiting α-KG-dependent enzymes, among which the members of JmjCs histone demethylases [22].

**Fig. 2:**
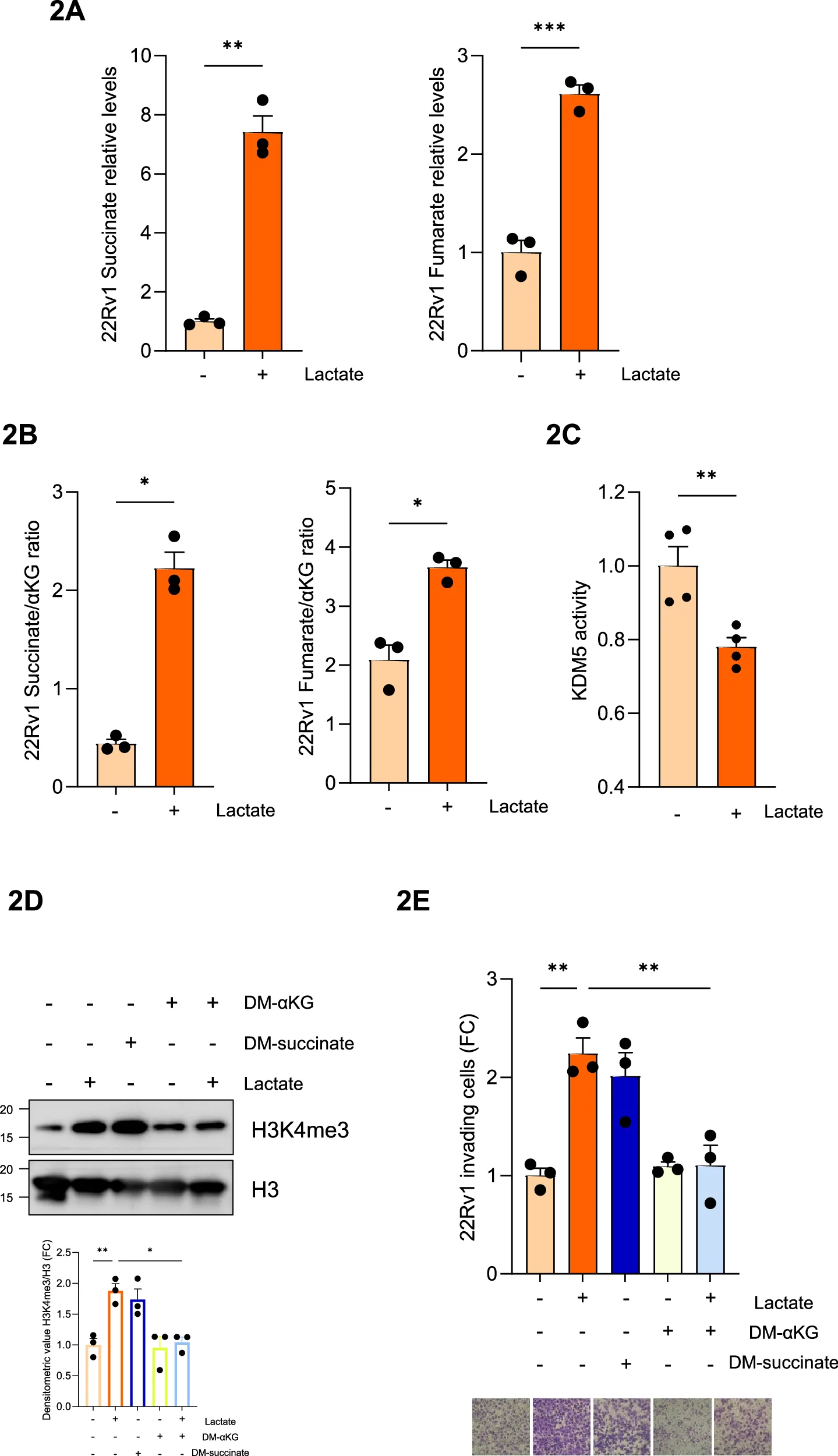
LA-sustained succinate/α-KG unbalance promotes H3K4me3-dependent PCa cell invasiveness. **A** GC-MS quantification of succinate and fumarate levels in 22Rv1 cells treated with or without LA (20 mM, 48 h). **B** Ratios of succinate/α-KG (left) and fumarate/α-KG (right) derived from GC-MS peak area analysis in 22Rv1 cells treated with LA. **C** KDM5 activity assay in 22Rv1 cells treated with or without LA (*n* = 4). **D** H3K4me3 levels evaluated by WB analysis in 22Rv1 cells treated with LA, cell-permeable dimethyl-succinate (DM-succinate, 5 mM) or dimethyl-αKG (DM-αKG, 3 mM). H3 was used as loading control. Densitometric analysis of H3K4me3/H3 ratio is shown (*n* = 3). **E** Invasion assay performed on 22Rv1 cells treated as reported in figure (**D**). Representative pictures are shown, magnification 20x. Data are represented as means ± SEM of *n* = 3 biological replicates unless otherwise stated. Statistical significance was assessed using unpaired two-tailed t-test (**A**–**C**) or one-way ANOVA with Tukey’s multiple comparisons test (E) (*P < 0.05; **P < 0.01; ***P < 0.001).

To investigate whether LA modulate intracellular TCA intermediates levels, we performed a GC-MS metabolomics analysis on 22Rv1 cells treated or not with LA. We observed an increase in intracellular succinate and fumarate in 22Rv1 cells exposed to LA compared to the untreated cells (Fig. 2A). Notably, calculation of fumarate- and succinate-to-αKG ratios revealed a marked increase in both metabolites upon LA treatment (Fig. 2B, S1D), indicating that LA drives a metabolic rewiring potentially affecting the demethylation profile of 22Rv1 cells. Accordingly, LA treatment led to a reduction of the H3K4me3-related demethylase (i.e., KDM5) activity in 22Rv1 cells (Fig. 2C).

To confirm the involvement of the LA-driven succinate in regulating H3K4 trimethylation mark in PCa cells, we mimicked the LA-boosted metabolic effect through the administration of cell permeable dimethyl (DM)-succinate. We observed increased H3K4me3 levels in 22Rv1 cells treated with DM-succinate (Fig. 2D). Conversely, administering DM-αKG (increasing intracellular levels of α-KG, acting as a cofactor for demethylases [22, 28]) to compete with LA-promoted succinate dampens the LA-induced increase in H3K4me3 levels (Fig. 2D). Coherently, DM-succinate and DM-αKG treatment phenocopied and prevented, respectively, the pro-invasive behaviour elicited by LA exposure in PCa cells (Fig. 2E, S1E).

Together, these data suggest that the LA-mediated succinate-to-α-KG unbalance is critical for the modulation of H3K4me3 mark enrichment in PCa cells.

### Impairment of H3K4me3 by OICR-9429 reduces LA-driven invasiveness in PCa

To target LA-induced global effects on histone H3K4 trimethylation, we employed OICR-9429, a selective compound able to suppress H3K4me3 and efficiently used to dampen tumor progression across multiple cancer models [29–31].

To dissect the H3K4me3-dependent transcriptional profile in 22Rv1 cells, we performed RNA-seq analysis on LA-exposed cells subjected or not to OICR-9429-mediated inhibition, to specifically identify genes modulates *via* H3K4me3. Treatment with OICR-9429 led to a marked reduction in H3K4me3 levels (Fig. S2A, S2B).

**Fig. 3:**
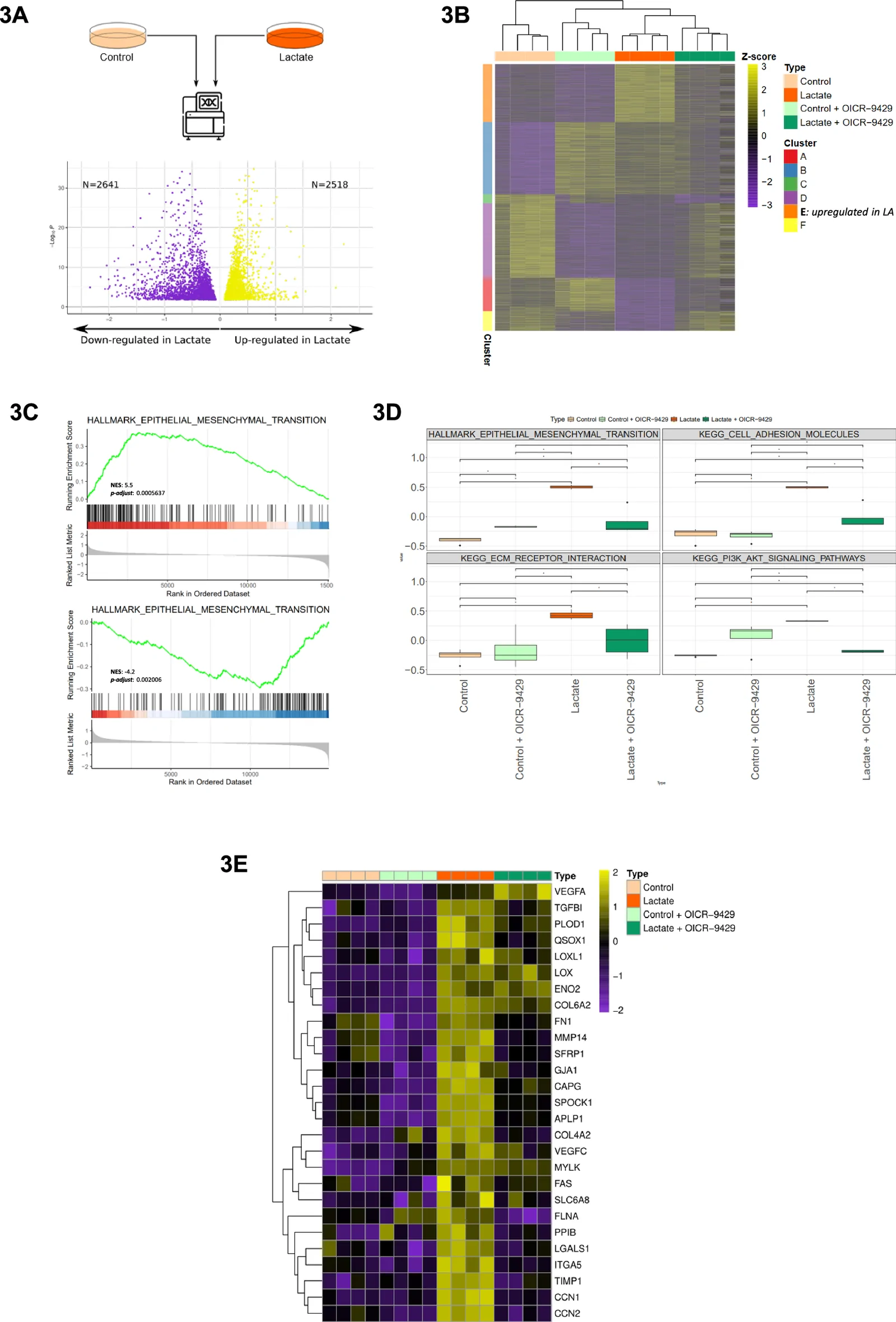
Impairment of LA-sustained H3K4me3 modification by OICR-9429 reduced LA-induced motility transcriptional programs. **A** Schematic overview of gene selection and analysis workflow. Differentially expressed genes (DEGs) were first identified by comparing LA-treated and control conditions. These DEGs were subsequently examined under OICR-9429 treatment. **B** Heatmap showing the expression patterns of selected genes across four experimental conditions: control ± OICR-9429, and LA ± OICR-9429. Genes and samples were hierarchically clustered using Euclidean distance for both rows and columns, with the complete linkage method, to group similar expression patterns. This clustering resulted in the identification of six distinct clusters, highlighting differential responses to LA and OICR-9429 treatments. Colour intensity represents relative gene expression levels in z-score. **C** GSEA plots illustrating the enrichment of the EMT pathway under two experimental comparisons: LA vs. Control (*upper* panel) and LA + OICR-9429 vs LA (*lower* panel). Each plot displays the ranked distribution of EMT-related genes based on differential expression, with enrichment scores reflecting the degree of coordinated upregulation or downregulation of the EMT gene set in the respective conditions. **D** Boxplots illustrating GSVA enrichment scores for selected biological pathways across experimental conditions. Each box represents the distribution of pathway activity within a condition, with individual data points reflecting enrichment scores for specific samples. **E** Heatmap showing the expression patterns of EMT-selected genes across four experimental conditions: control, control + OICR-9429, LA, and LA + OICR-9429. RNA-seq was performed on *n* = 4 biological replicates. Statistical significance was determined using p adjusted by FDR (**C**) or Wilcoxon rank-sum test (**D**) (*P < 0.05; **P < 0.01; ***P < 0.001).

Differentially expressed genes (DEGs) were initially identified by comparing LA-treated cells with control conditions (Fig. 3A, Suppl. table 1).

We focused on the cluster of genes upregulated by LA and restored to baseline levels upon co-treatment with OICR-9429, consistent with H3K4me3-dependent regulation (Fig. 3B, cluster E). This cluster comprised 1,124 genes, corresponding to 44.6% of all LA-upregulated DEGs.

We then performed hierarchical clustering of gene expression across all experimental conditions, and defined six distinct clusters associated with differential transcriptional responses to LA and OICR-9429, consistent with distinct regulatory effects induced by metabolic and epigenetic perturbations. We focused on the cluster of genes upregulated by LA and restored to baseline levels upon co-treatment with OICR-9429 (cluster E), consistent with H3K4me3-dependent regulation (Fig. 3B, Suppl. table 2). This cluster comprised 1,124 genes, corresponding to 44.6% of all LA-upregulated DEGs.

Gene Set Enrichment Analysis (GSEA) was first used to identify biological processes significantly enriched in LA-treated cells compared to controls. Among the significantly enriched pathways (Fig. 3C, S2C–E), the epithelial mesenchymal transition (EMT) signature emerged as particularly prominent in LA-treated cells (Fig. 3C), an effect that was reversed by OICR-9429 treatment, indicating that inhibition of H3K4me3 counteracts this transcriptional program. Notably, other enriched pathways were also modulated by OICR-9429 treatment, further supporting the role of H3K4me3 in regulating the transcriptional landscape induced by LA (Fig. S2C–E).

To further explore condition-specific modulation of gene expression signatures, we used Gene Set Variation Analysis (GSVA) to calculate the enrichment scores of selected gene expression signatures related to cell motility. Notably, inhibition of H3K4me3 reverted the LA-induced enrichment of signatures associated with extracellular matrix (ECM) organization and cell migration, including *EMT, Cell Adhesion, ECM-Receptor Interaction* and *PI3K-AKT Signaling* (Fig. 3D), all ones functionally linked to cell motility and invasive behaviour. Consistent with the transcriptomic profile, OICR-9429 treatment reduced the invasion ability of PCa cells (Fig. S2F, S2G).

Focusing on EMT signature, we found several genes involved in matrix remodelling and motility, like *PLOD1, SPOCK1, TGFI, QSOX1, LOX, LOXL1, FN1, MMP14, COL4A2, COL6A2* (Fig. 3E).

These findings demonstrate that LA-induced invasiveness in PCa cells is mediated by H3K4me3-dependent transcriptional programs, and that pharmacological disruption of this epigenetic mark through OICR-9429 transcriptionally impairs the pro-invasive phenotype.

### PLOD1 is functionally required for LA-induced invasion

Based on our previous findings indicating LA as a crucial regulator of de novo collagen synthesis pathway [15], we focused on PLOD1, an enzyme involved in the collagen synthesis and ECM remodelling [32, 33], both key processes strictly associated with cell invasiveness in several tumor models [15, 33, 34]. Compared with several other ECM- and motility-related genes emerging from the GSVA analysis, PLOD1 remains relatively underexplored in PCa, making it a particularly informative candidate for mechanistic validation. We prioritised PLOD1 because of its relevance in linking lactate exposure to ECM remodelling, consistent with our previous findings showing that lactate is an important regulator of collagen remodelling in PCa [15]. We investigated PLOD1 as one of the potential downstream gene of H3K4me3-mediated transcriptional responses in LA-treated cells.

**Fig. 4:**
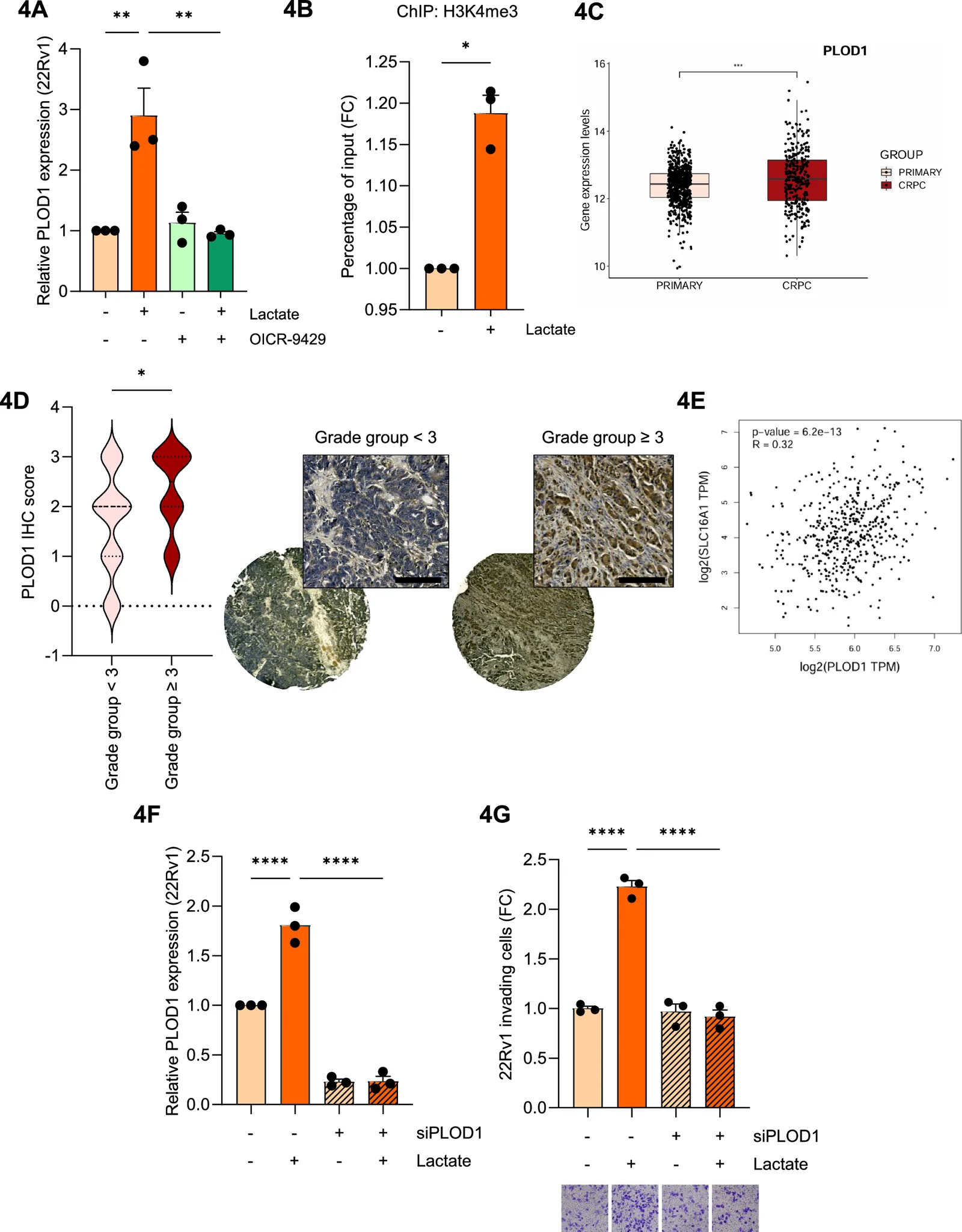
PLOD1 is a H3K4me3 target induced by LA and associated with invasive skills in PCa. **A** qRT-PCR analysis of PLOD1 mRNA levels in 22Rv1 cells treated with LA with or without OICR-9429. **B** ChIP-qPCR analysis of H3K4me3 enrichment at the PLOD1 promoter in 22Rv1 cells under the indicated conditions, shown as percentage of input. **C** PLOD1 expression levels in CRPC patients (*n* = 316) *vs* primary tumors (*n* = 714). **D** IHC analysis of PLOD1 protein expression in a PCa TMA stratified by *Grade Group* (low-grade, Grade Group < 3; high-grade, Grade Group ≥ 3; *n* = 46). Representative images are shown; scale bar: 20 μm. **E** Correlation analysis between *PLOD1* and *SLC16A1* (gene encoding MCT1) expression in the TCGA-PRAD dataset (*n* = 492). Pearson correlation coeff (R = 0.32). Each dot corresponds to an individual specimen. **F** qRT-PCR analysis of PLOD1 mRNA levels in 22Rv1 PLOD1-silenced cells and treated ± LA. **G** Invasion assay performed on 22Rv1 cells treated with LA ± PLOD1 silencing. Representative pictures are shown, magnification 20x. Data are represented as means ± SEM of *n* = 3 biological replicates unless otherwise stated. Statistical significance was assessed using unpaired two-tailed t-test (**B**, **D**) or one-way ANOVA with Tukey’s multiple comparisons test (**A**, **F**, **G**) (*P < 0.05; **P < 0.01; ***P < 0.001; ****P < 0.0001). The public dataset analysis is derived from annotated clinical cohorts [62].

Firstly, we confirmed in silico data by assessing PLOD1 abundance in LA-exposed PCa cells upon treatment with OICR-942. Indeed, we observed a significantly reduced PLOD1 expression in the co-treated cells compared to the control (Fig. 4A, S3A). We used ChIP-qPCR assays to further confirm that H3K4me3 was enriched at the PLOD1 promoter, proving a direct link between H3K4me3 and PLOD1 expression (Fig. 4B). These results suggest that the activation of PLOD1 might be involved in the H3K4me3-mediated PCa promotion.

To evaluate the clinical relevance of PLOD1 in PCa progression, we analyzed transcriptomic data from the TCGA prostate adenocarcinoma (PRAD) cohort, revealing significantly higher *PLOD1* expression in PCa tissues compared to normal tissues (Fig. S3B), indicating a strong correlation with the malignant disease. Moreover, *PLOD1* levels were even more elevated in castration-resistant prostate cancer (CRPC) relative to the primary tumors (Fig. 4C), further suggesting an association with disease progression and aggressiveness.

We extended this observation at the protein level by performing IHC on a TMA composed of PCa specimens across different clinical stages. Notably, PLOD1 protein expression was markedly increased in high *grade group* tumors compared to the low g*rade group* counterparts (Fig. 4D), and showed a positive correlation with *SLC16A1* expression (Fig. 4E). These data support the idea that higher LA content (i.e., MCT1-harboring tumor cells), promoting a H3K4me3-rich tumor environment, likely increases PLOD1 levels.

To functionally confirm the role of PLOD1, we performed siRNA-mediated knockdown of PLOD1 in PCa cells (Fig. 4F, S3C, S3D) to test its contribution to LA-driven invasive phenotype. Interestingly, PLOD1 silencing effectively impaired the motile features of LA-exploiting PCa cells (Fig. 4G, S3E), supporting a functional role in motility processes, further corroborating in silico data.

Altogether, these results outline a novel metabolic-epigenetic axis in which LA supports H3K4me3 modification to activate the transcription of key pro-migratory genes including PLOD1, offering new mechanistic insights driving PCa progression.

## Discussion

This study unveils a previously unappreciated metabolic-epigenetic axis linking LA to histone methylation. We demonstrated for the first time that elevated environmental LA levels selectively activate the H3K4me3 mark in PCa cell lines. These data are further supported by increased H3K4me3 levels in patient-derived high-grade PCa specimens and in LA-treated xenografted tumors, reinforcing the in vivo and clinical relevance of the here-reported LA-histone methylation mark. In line with our observations, a study on esophageal carcinoma revealed that H3K4me3 levels are significantly elevated in tumor tissues and strongly correlate with poor differentiation, advanced tumor stage, and reduced 5-year survival [35], confirming H3K4me3 as a relevant biomarker.

Our findings fit within the emerging view that cancer metabolism and epigenetic regulation are tightly interconnected, with metabolic states influencing chromatin by affecting the availability of substrates and cofactors, and modulating the activity of chromatin-modifying enzymes [2]. In this framework, lactate is emerging not only as a metabolic by-product or signalling metabolite [11, 12, 14, 36], but also as an upstream regulator of chromatin state [9, 10, 16]. While LA has been previously found to impact other histone modifications such as acetylation and lactylation [7, 9, 10, 37, 38], its effect on histone methylation has never been described. Importantly, our findings indicate that this mechanism is distinct from lactylation, as it is mediated indirectly through metabolic rewiring rather than through a direct acyl-donor function of lactate. These histone modifications act as complementary chromatin programs that collectively contribute in promoting tumor progression and aggressive disease features [7, 9, 10, 16]. Mechanistically, this phenomenon appears to be driven by a metabolic imbalance involving key TCA cycle intermediates. We show that LA treatment enhances succinate accumulation, therefore decreasing the α-KG/succinate ratio, a metabolic condition known to inhibit α-KG-dependent histone demethylases, including those acting on H3K4me3. This aligns with previous findings showing that exogenous succinate can elevate levels of various histone marks, including H3K4me3, H3K4me1, H3K27me2, and H3K79me2, highlighting its broad epigenetic impact [22].

In our model, LA reduces KDM5 activity, a specific α‑KG‑dependent H3K4me3-specific demethylase whose activity has been demonstrated to depend on succinate and fumarate levels [23, 36, 39, 40]. KDM5B has been implicated in suppressing cell migration and invasion in triple-negative breast cancer [41]. In keeping, inhibition of KDM5 activity by LA may therefore stabilize H3K4me3 levels and contribute to the acquisition of a more invasive phenotype. Of note, administration of DM-succinate phenocopied the effect of LA, promoting H3K4me3 levels and cellular invasiveness. Similarly, TRAP1, a mitochondrial chaperone, promotes tumor progression by suppressing succinate dehydrogenase (SDH) activity, leading to succinate accumulation, resulting in HIF1α stabilization under normoxia [42].

Our data extend the concept of LA as a pro-invasive inducer, linking the acquisition of invasive skills to methylation-mediated transcription regulation (specifically through H3K4me3) and extending its known roles beyond metabolic fuelling, immune evasion, and stromal remodelling [43, 44]. Thus, our study places LA within the broader framework of metabolite-driven epigenetic regulation in cancer, identifying histone methylation as an additional layer through which LA can promote tumour aggressiveness. Indeed, transcriptomic analysis revealed a LA-dependent gene expression signature enriched for EMT, ECM remodelling, and PI3K-AKT signalling pathways underpinning cancer cell motility.

To specifically identify the functional relevance of H3K4me3 in mediating the pro-invasive phenotype induced by LA, we employed a pharmacological strategy using the small-molecule inhibitor OICR-9429. Notably, treatment of PCa cells with LA in the presence of OICR-9429 led to a marked reduction in LA pro-invasive signatures. This is in line with recent findings in pancreatic ductal adenocarcinoma (PDAC), where TGFβ-driven migration was shown to depend on H3K4me3-mediated activation of key motility regulators, including *SNAI1* and *SOX9* [45]. Similarly, in colorectal cancer (CRC), the H3K9me3 methyltransferase SUV39H1 has been linked to enhanced cell migration and lymph node metastasis. Its overexpression promotes tumor progression in vivo, leading to significantly reduced survival in mouse models [46].

Among the genes regulated by H3K4me3 in response to LA treatment, PLOD1, a lysyl hydroxylase essential for collagen maturation and ECM remodelling [32, 33, 47], emerged as one functionally relevant downstream effector within this epigenetic programme. PLOD family members (*PLOD1-3*) have been previously associated with tumor progression and metastatic behaviour across various malignancies [32, 33, 48–50]. Indeed, we demonstrate that PLOD1 expression is upregulated by LA, and its silencing abrogates LA-induced invasion. These findings align with previous reports linking PLOD1 to aggressive phenotypes in breast, gastric cancers, and glioblastoma (GBM) [33, 47, 51, 52]. Particularly, PLOD1 expression has been found highly expressed in breast cancer, independent of estrogen receptor (ER) status, and correlated with tumor grade, suggesting a role in malignancy [51]. Likewise, in GBM, PLOD1 promotes tumor cell proliferation, migration, mesenchymal transition, and resistance to apoptosis. Furthermore, hypoxic conditions were found to exacerbate PLOD1-mediated tumor-promoting effects, consistent with the adaptive features of the GBM microenvironment [33]. The significance of PLOD1 upregulation in PCa is supported by recent studies showing that increased collagen deposition induced by LA (*via* P4HA1) actively promotes invasive behaviour and stem-like properties in head and neck squamous cell carcinoma and PCa; notably, in PCa these effects also involve discoidin domain receptor 1 (DDR1) [15, 53], highlighting the critical role of ECM remodelling in driving tumor aggressiveness. PLOD1 was particularly relevant because it directly controls lysyl hydroxylation, a crucial step for collagen maturation and stabilization, and because its role in PCa is still comparatively underexplored. This makes PLOD1 a useful candidate through which lactate-induced H3K4me3 rewiring may be translated into a pro-invasive extracellular matrix phenotype.

Importantly, our current findings on the H3K4me3-PLOD1 axis extend and complement previous observations. Rather than representing alternative mechanisms, the DDR1-P4HA1 [15], and the H3K4me3-PLOD1 axis are more likely to act in parallel, targeting distinct but functionally convergent steps of collagen maturation. While DDR1-P4HA1 signalling promotes collagen prolyl hydroxylation, the pathway described here supports lysyl hydroxylation through PLOD1 upregulation, thereby contributing to collagen stabilization and ECM remodelling. In this view, LA emerges as a multifaceted regulator of collagen processing, able to enhance the pro-invasive phenotype of PCa cells through coordinated and not-mutually exclusive effects on collagen-modifying pathways. Although the present study does not establish which axis is dominant, our data support a model in which both mechanisms coexist in driving ECM-dependent tumour aggressiveness, with LA enhancing collagen remodelling not only through previously described metabolic and extracellular mechanisms, but also at the transcriptional level through an H3K4me3-dependent programme.

In line with this, our data also showed elevated PLOD1 expression in high-grade PCa, supporting its potential as a biomarker of tumor aggressiveness and a candidate for targeted therapeutic intervention, although its contribution likely occurs within a broader LA-H3K4me3-driven transcriptional program rather than as the sole mediator of the pro-invasive phenotype.

Taken together, these findings add a novel layer to our understanding of LA as an oncometabolite, highlighting its previously underappreciated role in controlling histone methylation (i.e., H3K4me3). Beyond its established roles in lactylation and acetylation, LA may promote a pro-invasive phenotype by altering metabolite balance and reinforcing H3K4me3-dependent transcriptional programmes. These findings identify histone methylation as an additional epigenetic vulnerability that may be therapeutically relevant in PCa and, potentially, in other malignancies characterized by high LA burden and aggressive behaviour.

## Materials and methods

### Cell lines and reagents

Human PCa cell lines (22Rv1 [RRID:CVCL_1045], DU145 [RRID:CVCL_0105], PC3 [RRID:CVCL_0035] and LNCaP [RRID:CVCL_0395]) were obtained from the American Type Culture Collection (ATCC). All cells were maintained in DMEM (#ECB7501LEuroclone) supplemented with 10% FBS (#ECB4004L; Euroclone), 2 mmol/L L-glutamine and 1% penicillin/streptomycin. All cell lines were maintained at 37°C and 5% CO2 and were routinely tested for Mycoplasma contamination with the MycoAlert Mycoplasma Detection kit (#LT07–318; Lonza).

Lactic acid (LA) (#L1750, Sigma-Aldrich) was dissolved in water and used at 20 mM. Methyl-succinate (#S0755; Sigma-Aldrich) was dissolved in ethanol and used at a final concentration of 5 mM. Cell-permeable α-KG (dimethyl (DM) 2-oxoglutarate; Sigma-Aldrich) was used at a concentration of 3 mM. The MCT1 inhibitor AR-C155858 (#HY-13248; MedChemExpress, Sweden) was used at 40 μM in cell culture models. The WDR5-MLL inhibitor OICR-9429 (#HY-16993, MedChemExpress) was used at a final concentration of 10 μM.

### RNAi transfection and cell treatments

PCa cell lines were seeded into 6-well plates (150.000 per well/plates), to achieve 70% confluence the following day, when cells were transfected with 20 nM siRNA pool targeting PLOD1 (#EHU009851; Sigma-Aldrich), or the respective negative controls (#SIC001; siCTR; Sigma-Aldrich) using Lipofectamine RNAi-MAX Reagent (#13778-150; Thermo Fisher Scientific) and Opti-MEM (#31985062; Thermo Fisher Scientific) according to manufacturer’s instructions.

Alternatively, cells were treated with LA 20 mM and/or iMCT1 40 μM, OICR-9429 10 μM, DM-Succinate 5 mM, DM- α-KG 3 mM. The functional analyses were performed 48 h after treatments as described in the figure legends.

### Stromalized PCa model

The establishment of the 3D in vitro PCa cell model [27] was performed in poly-HEMA-coated six-well plates to prevent cell adhesion, by incubating 22Rv1 epithelial PCa cells, alone (1 × 10^5^ cells) or in coculture with stromal cells (2 × 10^5^ WMPY cells), with gelatin scaffolds (2 mg/well). The 3D PCa model was cultured in DMEM supplemented with 2% FBS at 37 °C and 5% CO_2_ for 21 days, changing the medium every five days. 3D PCa cell cultures were collected and fixed in 4% neutral-buffered formalin for 24 h and then included in agarose 1% PBS solution (100 μL for each experimental condition), fixed overnight, processed, and embedded in paraffin (FFPE).

### RNA extraction and qRT-PCR

Total RNA was extracted using RNeasy Kit (#74104; Qiagen), and cDNA synthesis was performed using iScript cDNA Synthesis Kit (#1708891; Bio-Rad). qRT-PCR was carried out by CFX96 Touch Real-Time PCR Detection System (Bio-Rad) using TaqMan assays (Life Technologies). The following probes were used in this study: PLOD1 (Hs00609363_m1), and data were normalized on HPRT1 (Hs02800695_m1) or TBP (Hs00427620_m1).

### Western blot analysis

PCa were washed with PBS and lysed for 20 min on ice in RIPA lysis Buffer (#89900; Thermo Fischer Scientific), and samples were then boiled at 95 °C with 4x Laemmli Sample Buffer (#1610747; Bio-Rad). Protein concentration was measured by BCA Kit (#23227; Thermo Fisher Scientific). Samples (2.5 μg for histone marks) were loaded on 4–20% acrylamide precast SDS/PAGE gels (#456-8096; Bio-Rad) and then transferred onto a polyvinylidene difluoride membrane with a Trans-Blot Turbo Transfer Pack (#1704157; Bio-Rad). The membranes were activated with methanol and then incubated in 5% non-fat-dry milk with the appropriate antibodies overnight at 4 °C. The following antibodies were used in this study: H3K4me3 (1:1000, #9751; Cell signaling), H3K36me3 (1:1000, #4909; Cell signaling), H3K9me3 (1:1000, #13969; Cell signaling), H3K27me3 (1:1000, #9733; Cell signaling), total histone H3 (1:2000, #14269; Cell signaling), PLOD1 (1:1000, #29480-1-AP; Proteintech) and HSP90 (1:1000, #sc-69703; SantaCruz Biotechnology). Membranes were then incubated with the corresponding secondary antibodies for 1 h at room temperature and visualised with Amersham AI600 Chemiluminescent Imager (Amersharm, Heights, IL, USA).

### Chromatin immunoprecipitation (ChIP) assay and ChIP-qPCR

Chromatin immunoprecipitation (ChIP) experiments were performed by using Pierce Magnetic ChIP Kit (#26157; Thermo Fisher Scientific). Briefly, cells were cross-linked with 1% formaldehyde for 10 minutes followed by quenching with 0.25 M glycine for 5 min. Post-wash, 2 × 10^6^ cells were scraped and resuspended in 500 μL of membrane extraction buffer, incubated on ice for 10 min and centrifuged to separate the nuclei from the cytosolic fraction. Nuclei were resuspended in 300 μL of nuclear lysis buffer disrupted in a Q500 sonicator (Qsonica) at 4 °C (30 s on/off pulses, 10 cycles, 30% power). After verification on agarose gel of the right size of chromatin shearing (approximately 200–400 bp), the samples were centrifuged, and supernatant collected in a fresh tube after removal of 3% input samples. 90 μL of the chromatin solution were incubated with H3K4me3 antibody (1:100, #ab8580; Abcam), or corresponding IgG (1:100), overnight at 4 °C. Next day A/G magnetic beads were added, and incubation continued for an additional 2 h at 4 °C, followed by sequential washes. In parallel with the input samples, the eluted chromatin was then subjected to RNase treatment, followed by Proteinase K digestion for 1.5 h heated at 65 °C to reverse the crosslinking. DNA was recovered by purifying the samples on provided columns. The final DNA was eluted in 25 μL nuclease-free water for ChIP-qPCR. The primers used for evaluating PLOD1 promoter in SYBR-Green qPCR are forward 5’-CCCCATACCTCGGCCATG-3’ and reverse 5’-TTAAGTGGACAAGGAGCCGG-3’.

### Invasion assay

Invasion assay were performed using an 8-μm-pore transwell (#3428; Corning) coated with reconstituted Matrigel (#356334; BD Biosciences) to assess invasive capacity. Cells were first seeded in 6-well plates, and all reagents (LA 20 mM, iMCT1 40 μM, OICR-9429 10 μM, DM-Succinate 5 mM, DM- α-KG 3 mM) were added 24 h later. After 48 h of treatment, cells were harvested and seeded into the upper chamber in serum-free medium of the transwell at a density of 100.000 cells/well in serum-free medium, and allowed to migrate for 16 h toward complete medium. Air-dried membranes were fixed with methanol and stained with crystal violet 0.1% (#C0775; Sigma-Aldrich). Invasive potential was evaluated by counting the cells that migrated to the lower surface of the filters in four randomly selected fields.

### Histone Demethylase (HDM) activity assay

Nuclear proteins were extracted using the *nuclear extraction kit* (#ab113474; Abcam) and quantified with a BCA protein assay kit. Histone demethylase (H3K4) activity was assessed using the histone demethylase (KDM5) activity quantification assay kit (#ab113463; Abcam), following the manufacturer’s instructions. Briefly, 50 μL of diluted HDM substrate was added to each sample and control well. Sample wells received an additional 28 μL HDM assay buffer and 2 μL of nuclear protein (containing 4–20 μg of protein). Control wells received 30 μL of HDM assay buffer, while blank wells containing assay buffer only. The plate was incubated at 37 °C for 60 min. Following incubation, diluted capture antibodies were added to each well and incubated at room temperature with gentle shaking for 60 min. Subsequently, diluted detection antibodies were added and incubated for 30 min. Fluorescence development reagents were then added and incubated in the dark for up to 5 min. HDM activity (OD/min/mg) was calculated using the following formula and is reported in the graphs as fold change (FC) relative to the control:

$${HDM\; activity}=\frac{{\rm{Sample\; OD}}-{\rm{Blank\; OD}}}{{\rm{Reaction\; time}}\,\left(\min \right)\,x\,{\rm{Protein\; Amount\; added}}({\rm{\mu }}{\rm{g}})}x1000$$

### Metabolomics

For selected ion monitoring (SIM) mode MS analysis, cells were scraped in 80% methanol, and phase separation was achieved by centrifugation at 4 °C. The methanol-water phase containing polar metabolites was separated and dried using a vacuum concentrator. Dried polar metabolites were dissolved in 20 μL of 2% methoxyamine hydrochloride in pyridine (Pierce-Thermo Fisher) and held at 37 °C for 2 h. After dissolution and reaction, 80 μL N-tert-Butyldimethylsilyl-N-methyltrifluoroacetamide with 1% tert-Butyldimethylchlorosilane MBTSTFA + 1% TBDMCS (Thermo Fisher) was added, and samples were incubated at 60 °C for 60 min. Gas chromatographic runs were performed with helium as carrier gas at 0.6 mL/min. The split inlet temperature was set to 250 °C and the injection volume of 1 μL. The GC oven temperature ramp was from 70 to 280 °C. The first temperature ramp was from 70 to 140 °C at 3 °C/min. The second temperature ramp was from 140 to 180 °C at 1 °C/min. Finally, the latest temperature ramp was from 180 to 280 °C at 3 °C/min. For the Quadrupole, an EI source (70 eV) was used. The ion source and transfer line temperatures were set, respectively, to 250 °C and 290 °C. For the determination of relative metabolite abundances, the integrated signal of all ions for each metabolite fragment was normalized by the signal from norvaline and protein content. Additionally, the succinate/α-KG and fumarate/α-KG ratios were calculated to assess TCA cycle metabolite balance.

### RNA-seq data processing

The RNA-seq libraries were sequenced on paired-end 150 bp mode on NovaSeq 6000 platform (Illumina, San Diego, CA). The RNA-seq was analyzed using Transcriptomestic, a snakemake toolkit for RNAseq data analysis, publicly available at https://github.com/littleisland8/Transcriptomestic. Raw reads were quality-checked using FastQC (v 0.12.1) [54]. Adapter trimming and low-quality base removal were performed using fastp [55]. Reads were aligned to the reference genome GRCh38 available through the 1000 Genomes FTP site ftp://ftp.1000genomes.ebi.ac.uk/vol1/ftp/technical/reference/GRCh38_reference_genome/GRCh38_full_analysis_set_plus_decoy_hla.fa using STAR (v2.7.11a) [56] with default parameters. Aligned reads were quantified at the gene level using HTSeq-count (v2.0.2) [57] with annotations from GRCh38 Ensemble GTF file (v.111). Differential expression analysis was performed by DESeq2 (v.1.46.0) [58]. Gene expression signatures were estimated by the GSVA method implemented in the GSVA R package (v2.0.1) [59], using DESeq2’s rlog-transformed counts as input. Gene signatures were compared across sample groups using Wilcoxon rank-sum test.

Gene Set Enrichment Analysis (GSEA) was performed using the R package DOSE (v.4.0.1) [60]. Genes were ranked according to log2 fold change, and enrichment was assessed against curated gene sets from GO (CC, BP and MF), KEGG, REACTOME and from the MSigDB database (MSigDB v2024.1.Hs) including Hallmark, C2 (curated) and C6 (oncogenic signatures) [61]. Analyses were run using *n* = 1000 permutations, with gene set size thresholds set between min = 2 and max = 500 genes. Enrichment scores were normalized (NES), and statistical significance was determined using FDR with a threshold of 0.05 considered significant.

### Gene Set Variation Analysis (GSVA)

Gene Set Variation Analysis (GSVA) was performed using the GSVA package (v2.0.1) in R [59] to assess sample-wise enrichment of gene sets in an unsupervised manner. The input was the DESeq2’s rlog-transformed counts and analysis were run with the method = ‘gsva’. Gene sets used were the same as those employed in GSEA, and enrichment scores were compared across sample groups using Wilcoxon rank-sum test.

### In silico database analysis

A comprehensive database of PCa patients that includes 714 primary tumors and 316 CRPC cases was used [62]. VST-normalized expression data and its annotations were downloaded from the Zenodo repository (https://zenodo.org/). To compare *PLOD1* expression levels in primary samples and CRPC, the Wald test from DESeq2 package was performed on VST-normalized expression values. GEPIA2 was used to analyze *PLOD1* expression levels, as transcripts per million (TPM), between normal and tumor samples, as well as to perform correlation analysis between *PLOD1* and *SLC16A1* expression (as TPM) using Pearson correlation coefficient.

### In vivo experiment

Six to 8-week-old male SCID mice (NOD.CB17-Prkdcscid/ NCrHsd, Envigo) were housed and used at the Centro Stabulazione Animali da Laboratorio (CESAL, Florence, Italy) under sterile conditions with ad libitum access to food and water. For spontaneous metastasis assay, 1 × 10^6^ DU145 cells were resuspended in 100 μl PBS and subcutaneously injected into the flanks of SCID mice. Mice were randomly divided into two groups (*n* = 4) and treated or not with lactate (i.p.,1 g/kg/daily). Animal work was carried out under the Project license n. 591/2022-PR, and was approved by the Italian Health Minister. All the procedures were conducted in accordance with the protocols approved by the Institutional Animal Care and Use Committee of the University of Florence.

### Immunohistochemistry

Individual specimens from tumour xenografts were dissected and fixed in 4% formalin for histology and IHC analysis. For human PCa analysis, we used a tissue microarray (TMA) (#PR633a; DBA), containing 60 cases of prostate adenocarcinoma. Immune-staining of prostate TMA sections were performed using antibodies against H3K4me3 (1:500, #9751; Cell signaling) and PLOD1 (1:200, #SAB1301577; Sigma-Aldrich). H3K4me3 slides from tumor xenografts were developed with 3,3-diaminobenzidine (DAB) and counterstained with hematoxylin (#DS9800; Leica), dehydrated and mounted. IHC was performed using the Leica BOND-MAX automated system (Leica Microsystems, Italy). Staining score for H3K4me3 and PLOD1 was independently analysed by at least two researchers and given a different score, defined as 0 for negative, 1 for weak, 2 for moderate, 3 for strong. Tumors were stratified into low-grade (Grade Group < 3) or high-grade (Grade Group ≥ 3). All images were captured through a slide scanner (Aperio LV1; Leica Biosystems) and analysed using the software ImageScope (Leica Biosystems).

### Statistical analysis

Statistical analysis was carried out with GraphPad Prism v.10.6.0 (GraphPad Software, USA). Data are presented as means ± SEM from at least three independent experiments. Statistical comparisons: two-tailed unpaired Student’s t-test or one-way ANOVA followed by Tukey’s correction. Pearson’s correlation coefficient was used to assess gene co-expression. Statistical significance was considered when *P* < 0.05.

## Supplementary information

Figures (high resolution)

Supplementary figure and table legends

Supplementary table 1

Supplementary table 2

Uncropped original western blot

Figure S1.

Figure S2.

Figure S3.

## Data Availability

The data generated during the current study are available from the corresponding author upon request. The accession number for the RNA-seq data performed on 22Rv1 cells treated with LA ± OICR-9429 reported in this paper is GEO: *GSE309183*.
