# Supplementary material for: Lactate-induced H3K4me3 histone mark promotes PLOD1-driven prostate cancer invasion
Source: Cell Death Dis. 2026 Jul 7;17(1):843. doi: 10.1038/s41419-026-09091-4 (PMC13620169; doi:10.1038/s41419-026-09091-4)

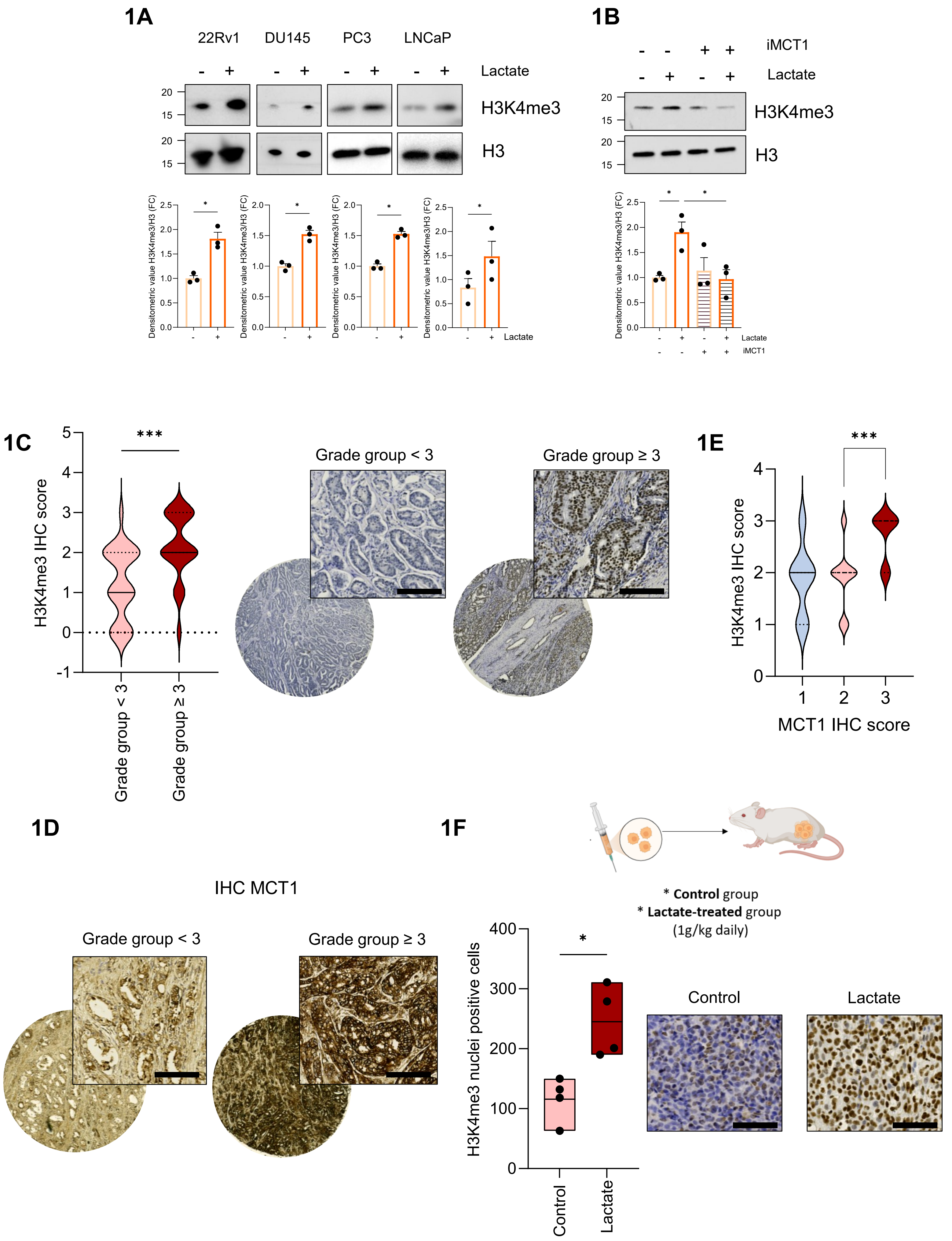
1A
22Rv1
DU145
PC3
LNCaP
- + - + - + - +
Lactate
H3K4me3
H3
20
15
Densitometric value H3K4me3/H3 (FC)
*
Lactate
1B
iMCT1
Lactate
H3K4me3
H3
Lactate
iMCT1
1C
H3K4me3 IHC score
***
Grade group < 3
Grade group ≥ 3
1E
H3K4me3 IHC score
***
MCT1 IHC score
1D
IHC MCT1
Grade group < 3
Grade group ≥ 3
1F
* Control group
* Lactate-treated group
(1g/kg daily)
H3K4me3 nuclei positive cells
*
Control
Lactate

2A

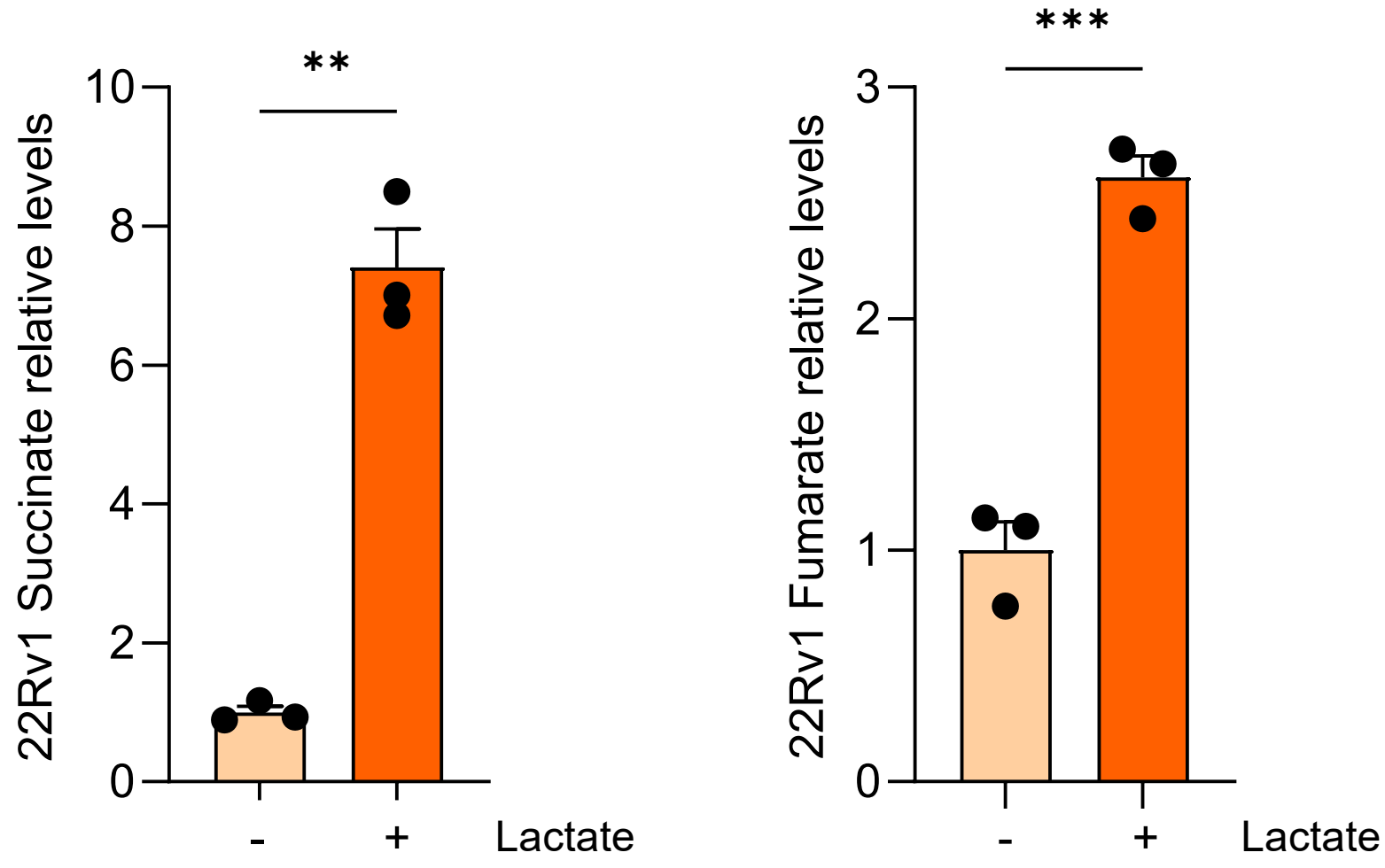

22Rv1 Succinate relative levels
22Rv1 Fumarate relative levels
**
***
-
+
Lactate

2B

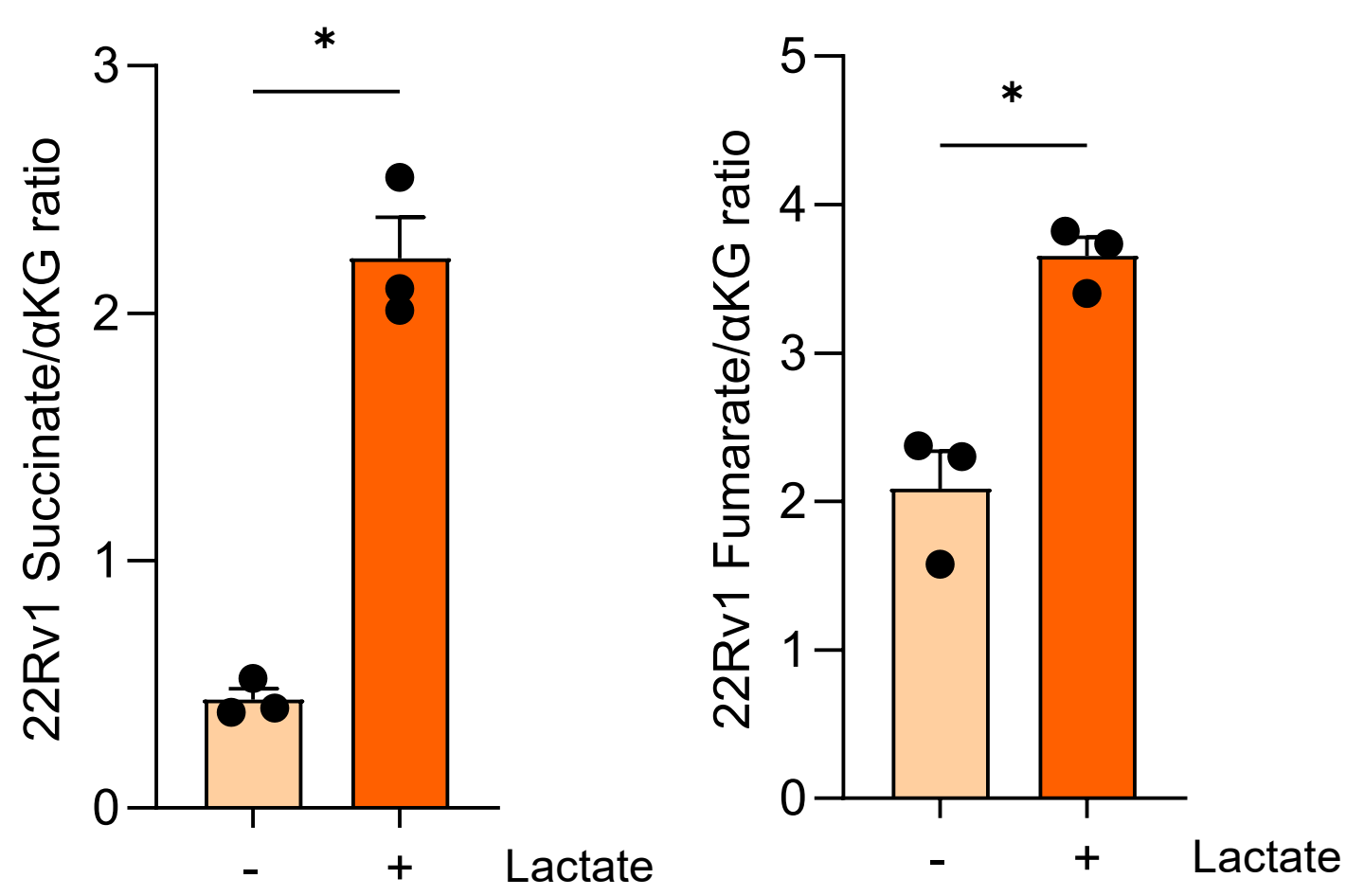

22Rv1 Succinate/αKG ratio
22Rv1 Fumarate/αKG ratio
*
-
+
Lactate

2C

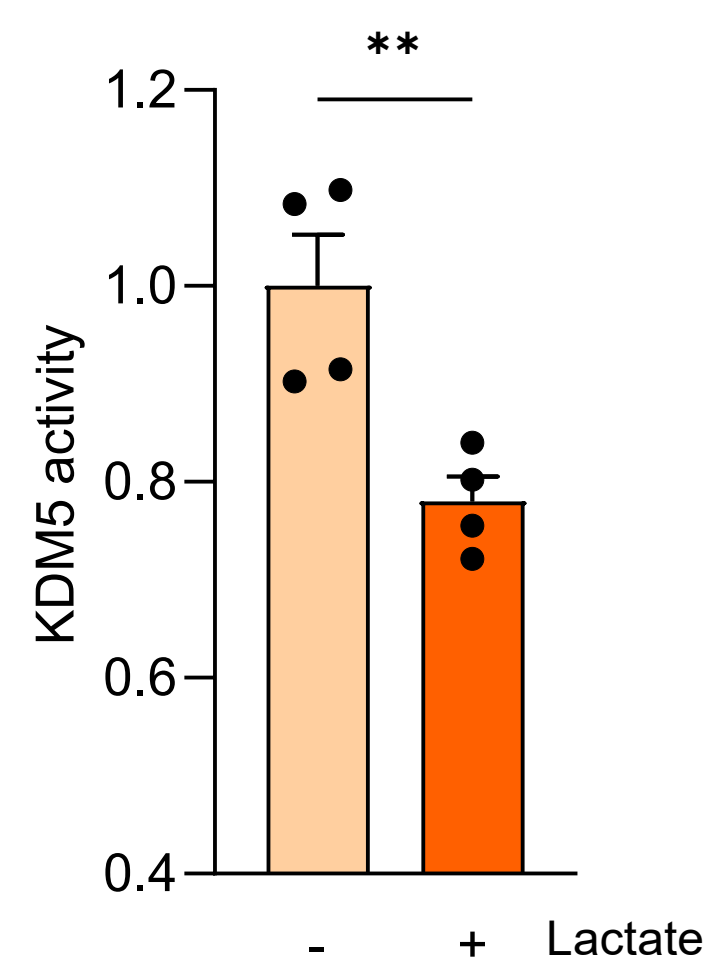

KDM5 activity
**
-
+
Lactate

2D

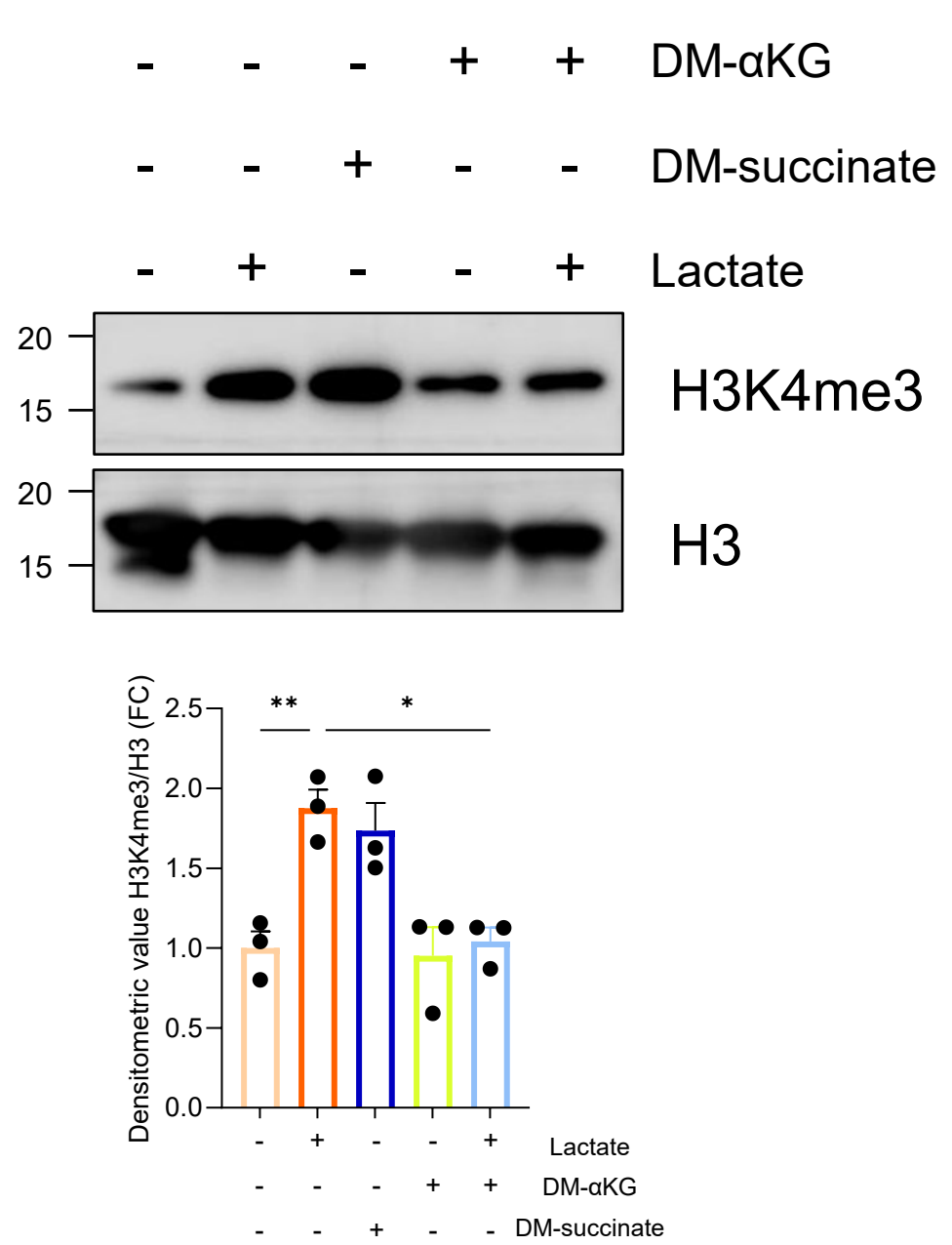

DM-αKG
DM-succinate
Lactate
H3K4me3
H3
Densitometric value H3K4me3/H3 (FC)

2E

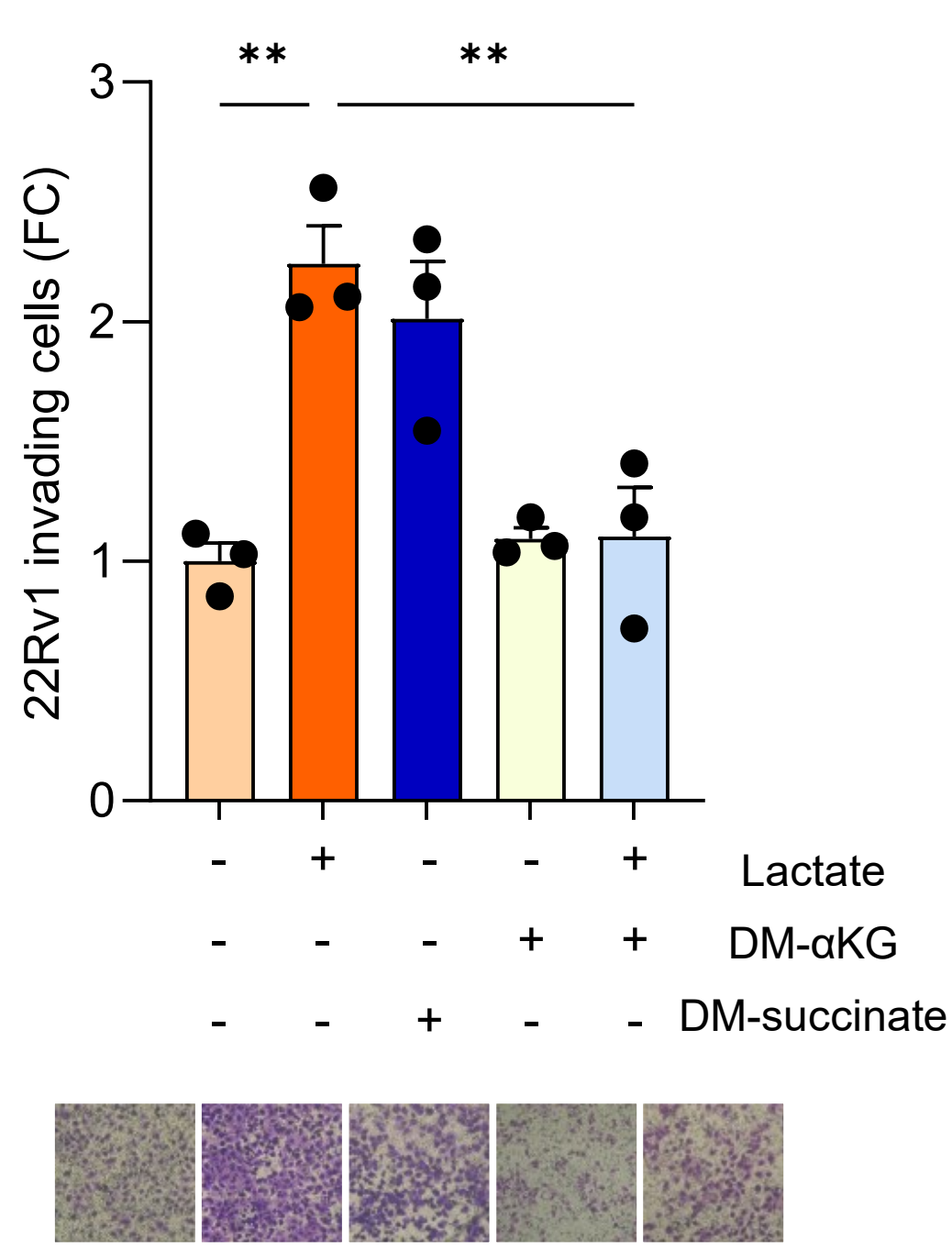

22Rv1 invading cells (FC)
**
Lactate
DM-αKG
DM-succinate

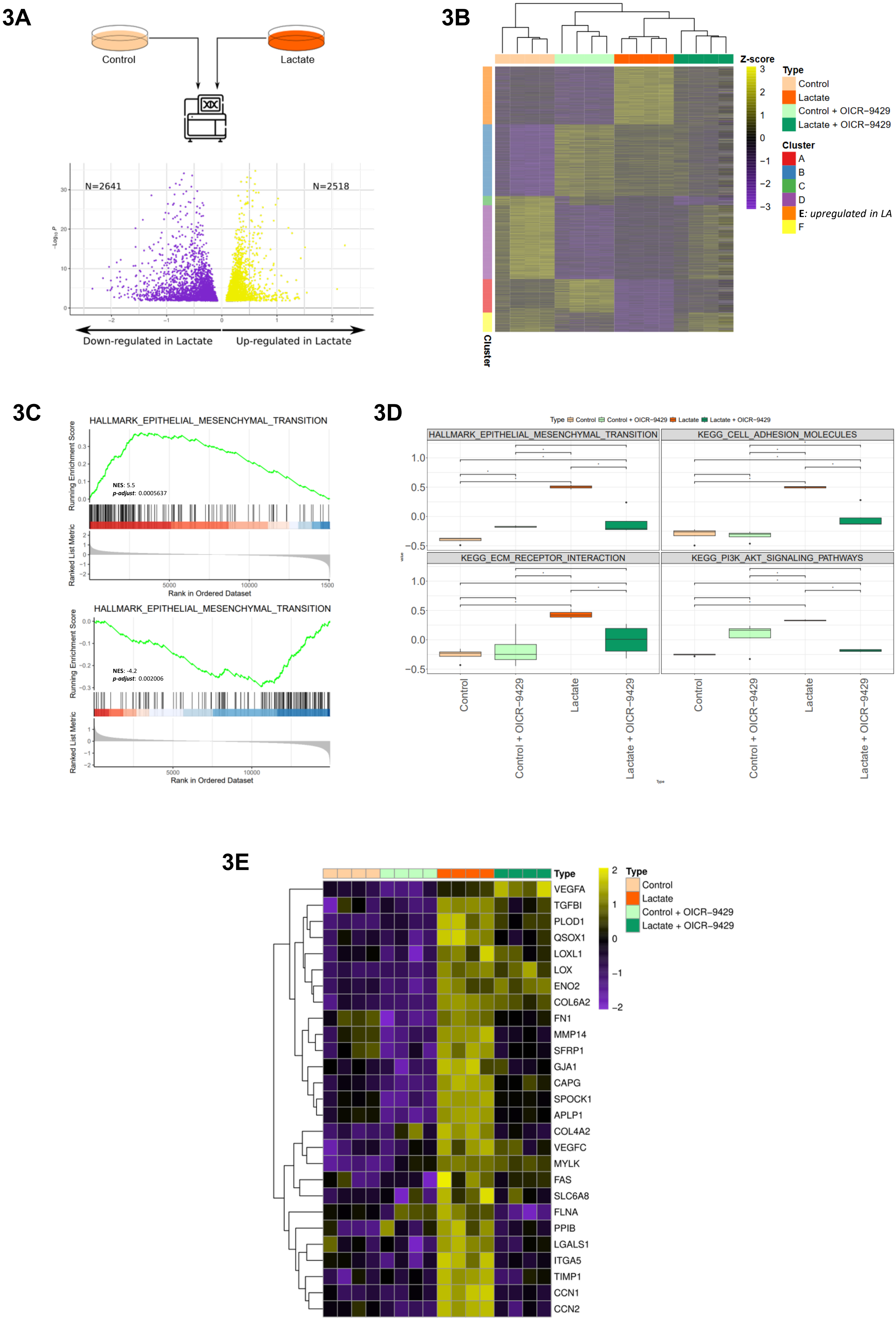
3A
Control
Lactate
N=2641
N=2518
Down-regulated in Lactate
Up-regulated in Lactate
3B
Z-score
Type
Control
Lactate
Control + OICR−9429
Lactate + OICR−9429
Cluster
A
B
C
D
E: upregulated in LA
F
3C
HALLMARK_EPITHELIAL_MESENCHYMAL_TRANSITION
NES: 5.5
p-adjust: 0.0005637
Running Enrichment Score
Ranked List Metric
Rank in Ordered Dataset
HALLMARK_EPITHELIAL_MESENCHYMAL_TRANSITION
NES: -4.2
p-adjust: 0.002006
Running Enrichment Score
Ranked List Metric
Rank in Ordered Dataset
3D
Type Control Control + OICR−9429 Lactate Lactate + OICR−9429
HALLMARK_EPITHELIAL_MESENCHYMAL_TRANSITION
KEGG_CELL_ADHESION_MOLECULES
KEGG_ECM_RECEPTOR_INTERACTION
KEGG_PI3K_AKT_SIGNALING_PATHWAYS
Control
Control + OICR−9429
Lactate
Lactate + OICR−9429
3E
Type
VEGFA
TGFBI
PLOD1
QSOX1
LOXL1
LOX
ENO2
COL6A2
FN1
MMP14
SFRP1
GJA1
CAPG
SPOCK1
APLP1
COL4A2
VEGFC
MYLK
FAS
SLC6A8
FLNA
PPIB
LGALS1
ITGA5
TIMP1
CCN1
CCN2
Type
Control
Lactate
Control + OICR−9429
Lactate + OICR−9429

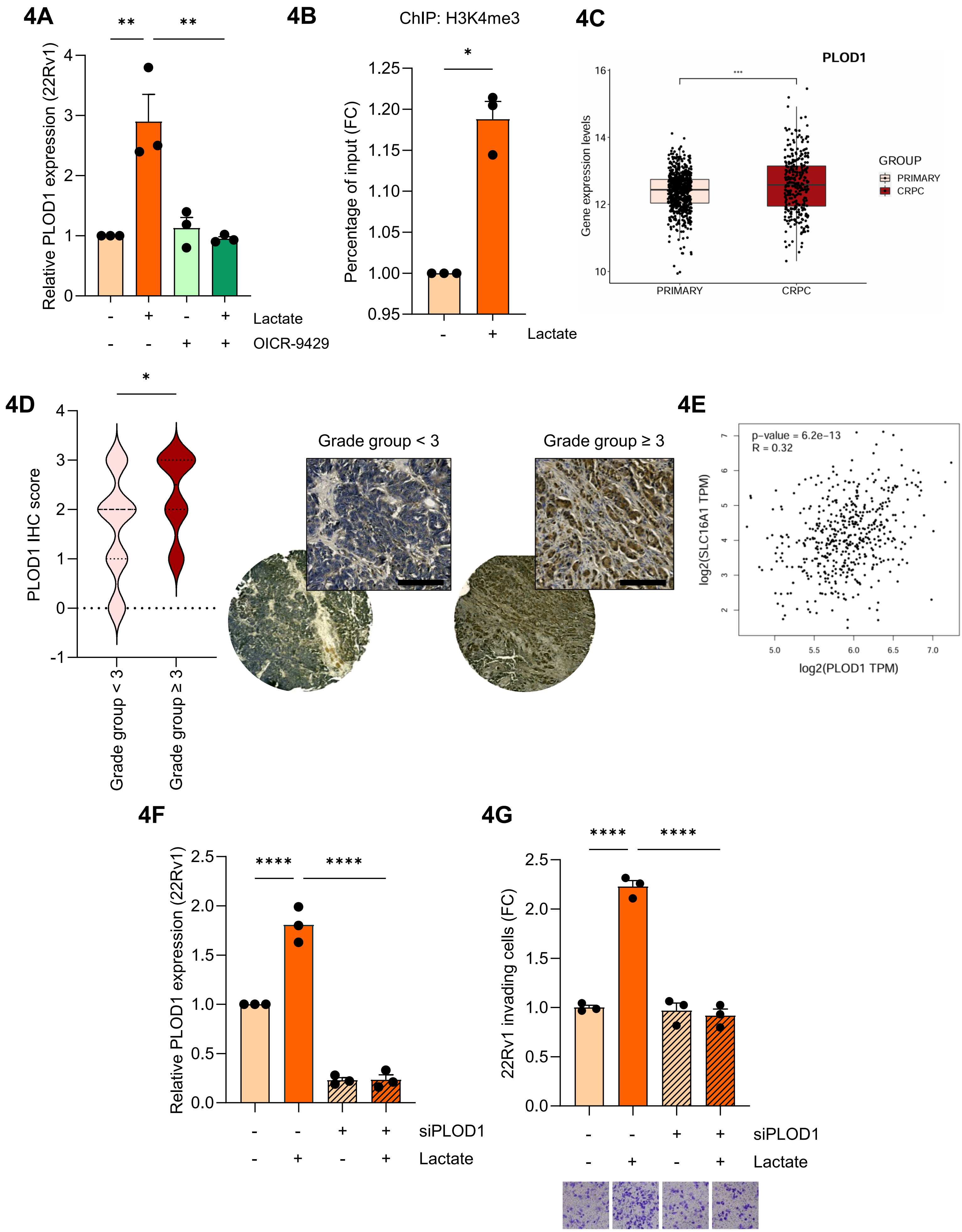
4A
**
**
Relative PLOD1 expression (22Rv1)
0
1
2
3
4
-
+
-
+
Lactate
-
-
+
+
OICR-9429
4B
ChIP: H3K4me3
*
Percentage of input (FC)
0.95
1.00
1.05
1.10
1.15
1.20
1.25
-
+
Lactate
4C
PLOD1
***
Gene expression levels
10
12
14
16
PRIMARY
CRPC
GROUP
PRIMARY
CRPC
4D
*
PLOD1 IHC score
-1
0
1
2
3
4
Grade group < 3
Grade group ≥ 3
Grade group < 3
Grade group ≥ 3
4E
p-value = 6.2e-13
R = 0.32
log2(SLC16A1 TPM)
2
3
4
5
6
7
5.0
5.5
6.0
6.5
7.0
log2(PLOD1 TPM)
4F
****
****
Relative PLOD1 expression (22Rv1)
0.0
0.5
1.0
1.5
2.0
2.5
-
-
+
+
siPLOD1
-
+
-
+
Lactate
4G
****
****
22Rv1 invading cells (FC)
0.0
0.5
1.0
1.5
2.0
2.5
-
-
+
+
siPLOD1
-
+
-
+
Lactate

S1A

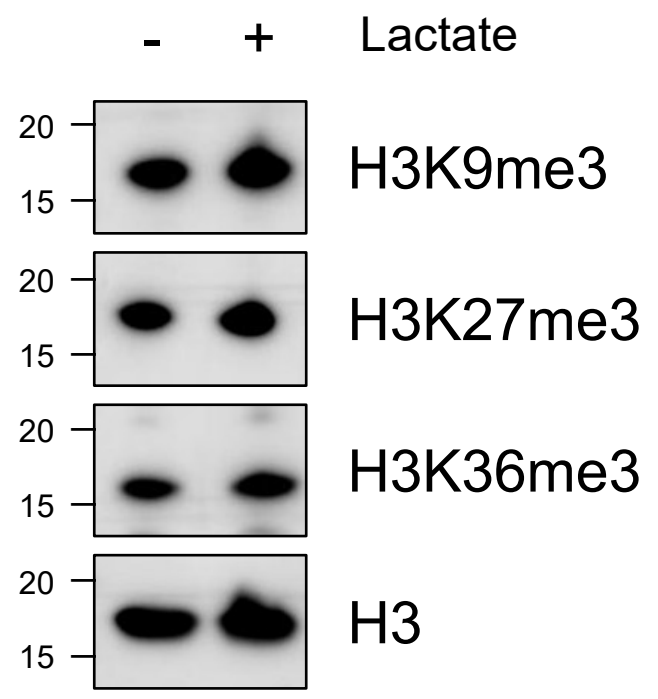

S1B

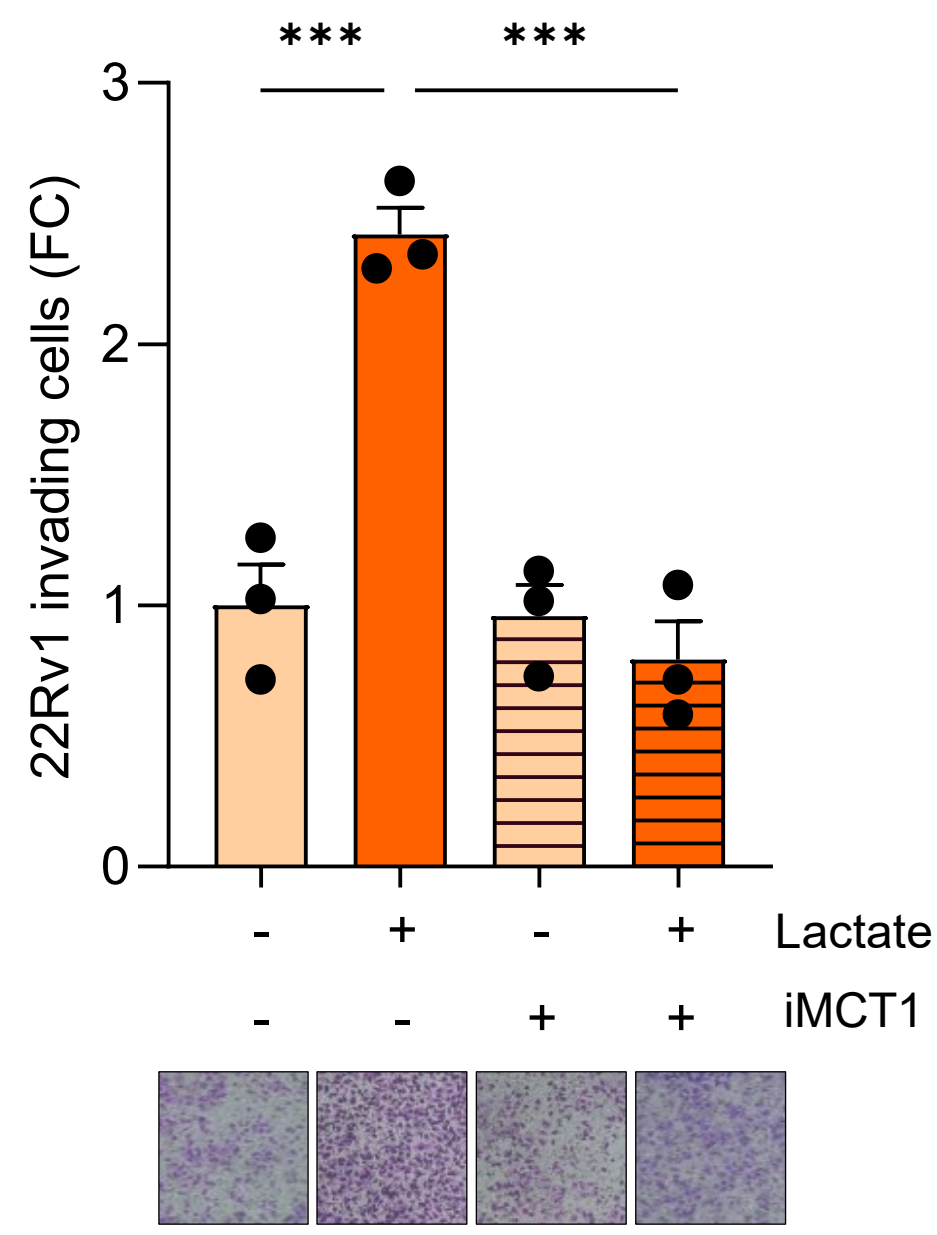

S1C

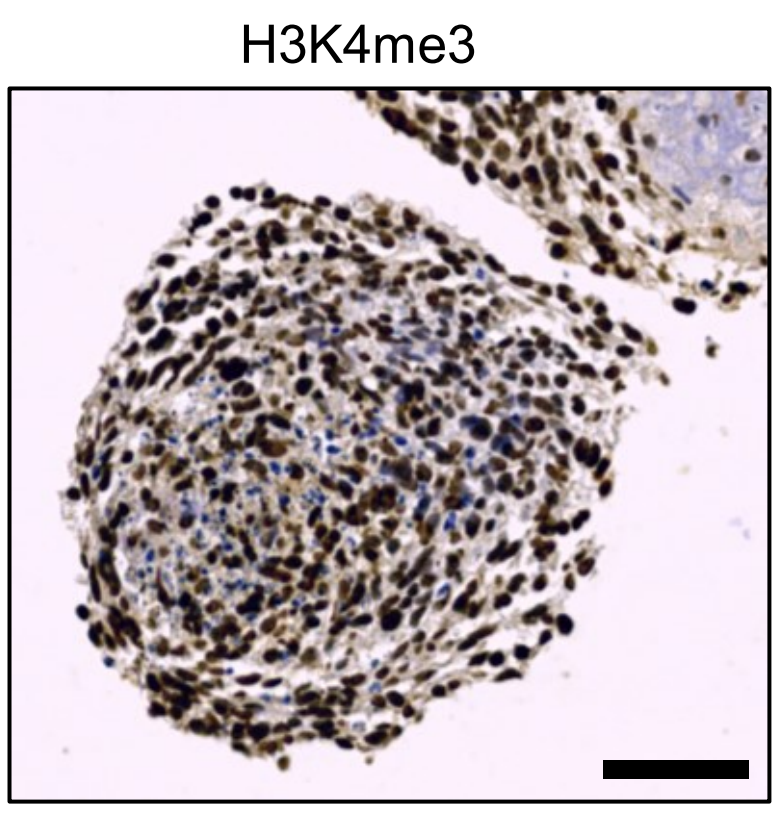

S1D

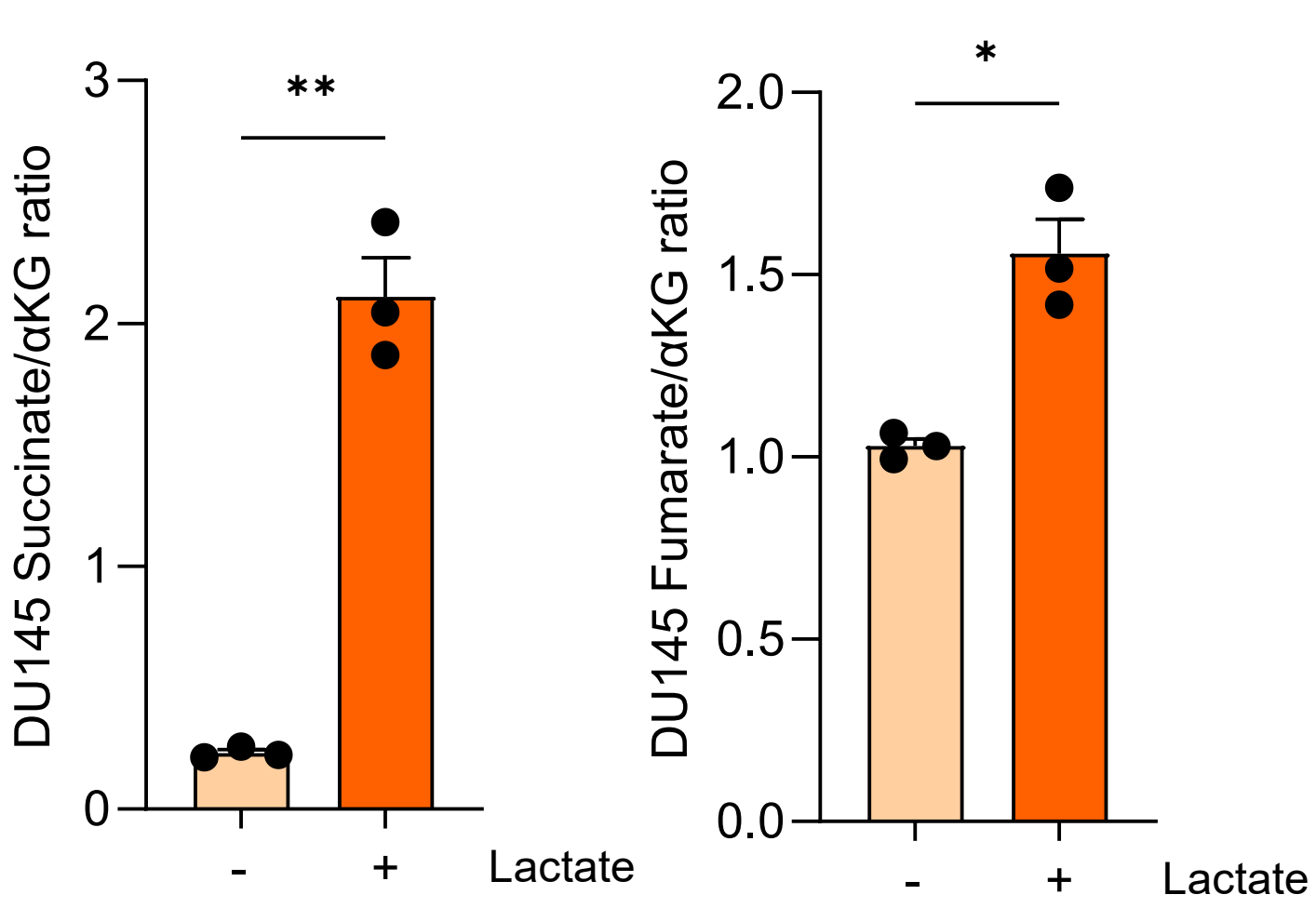

S1E

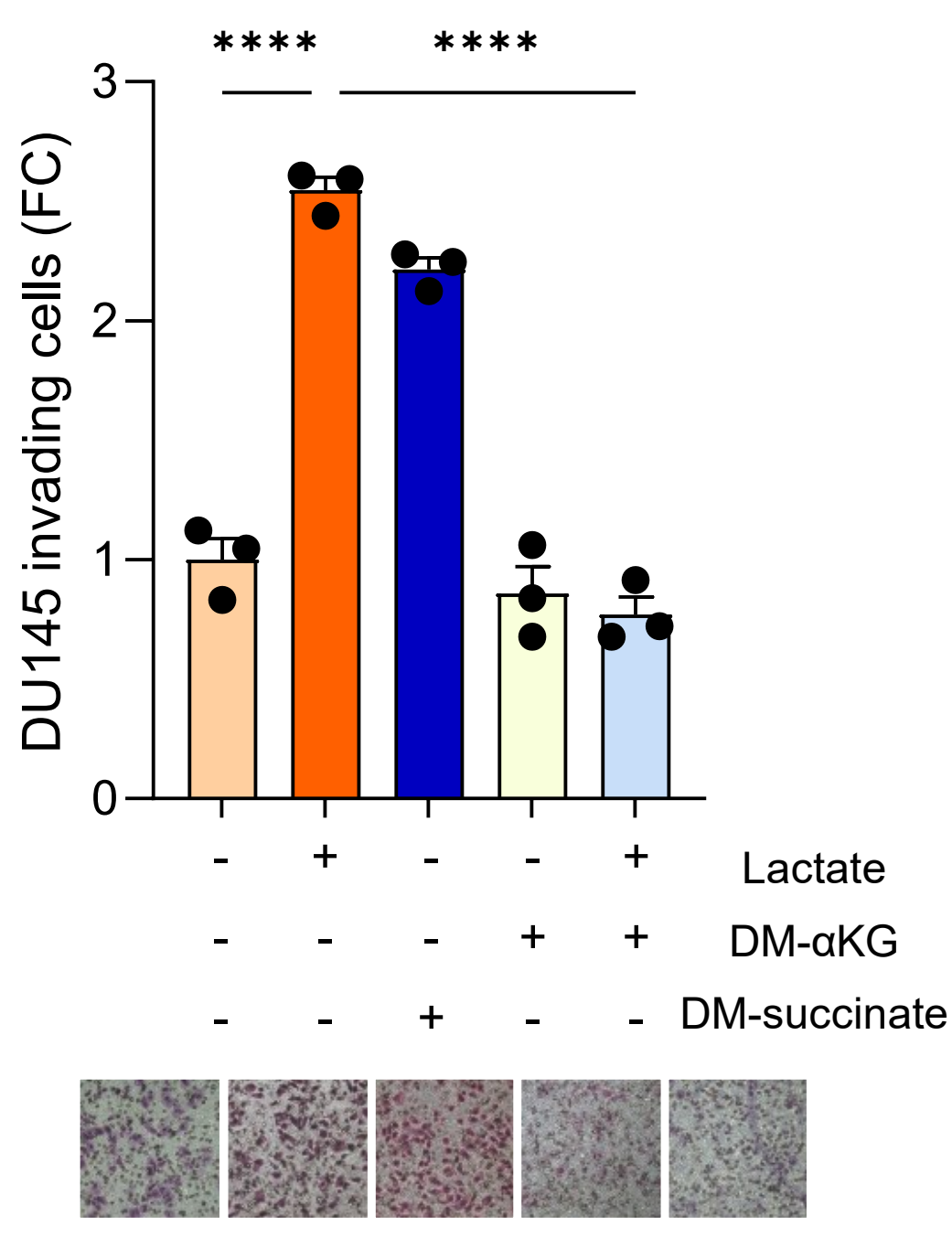

S2A

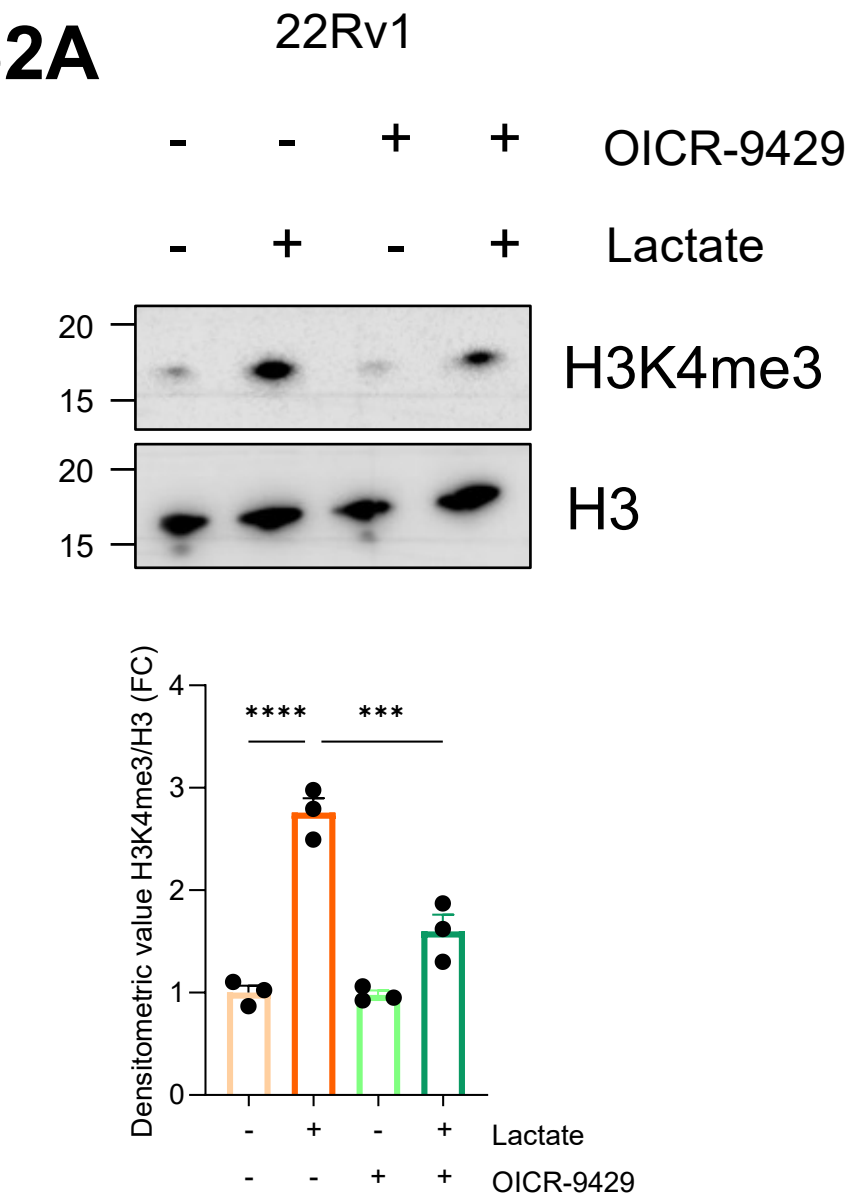

S2B

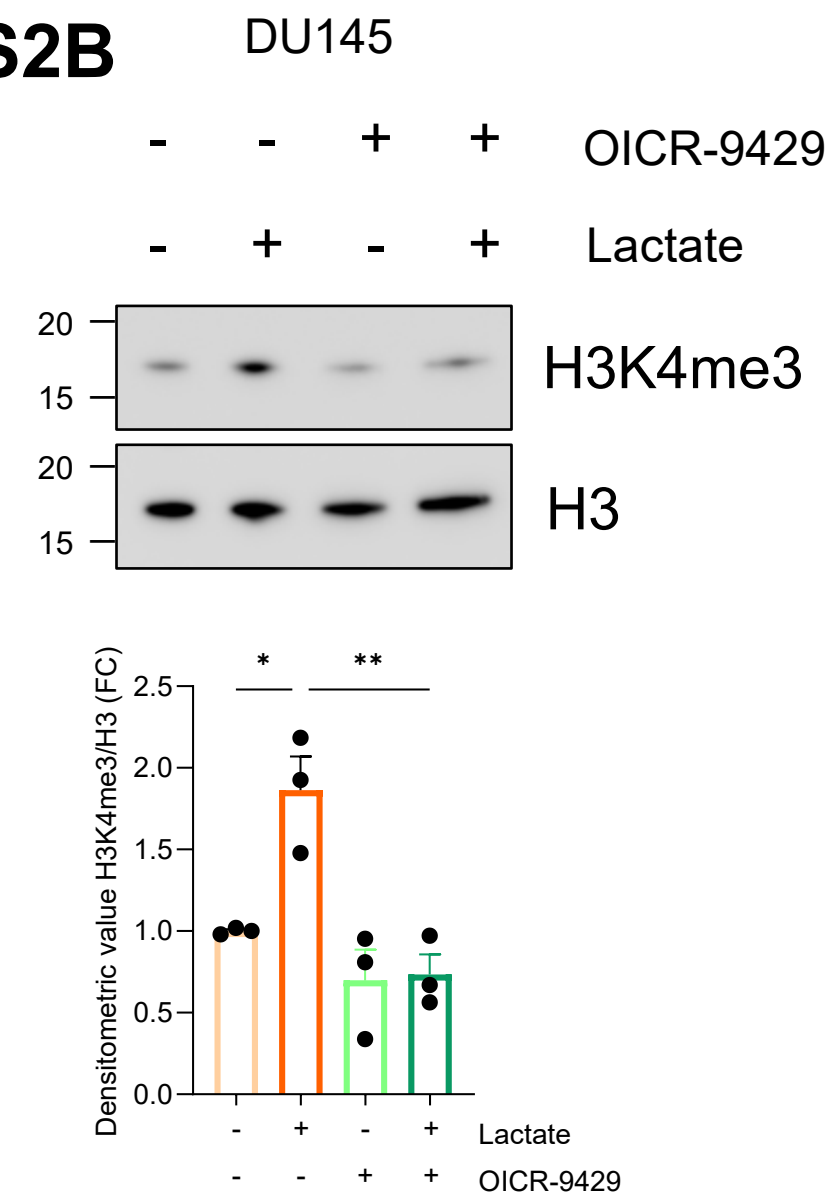

S2C

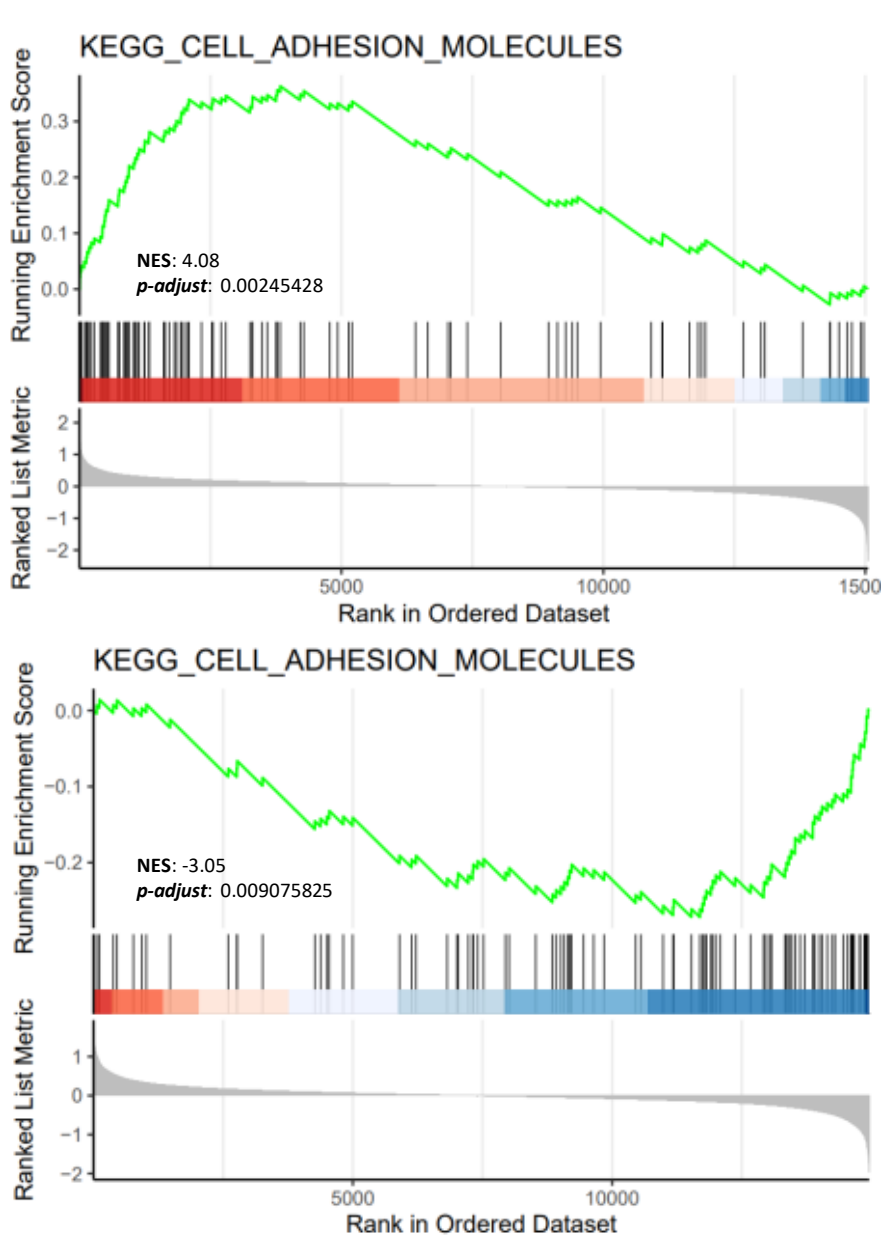

S2D

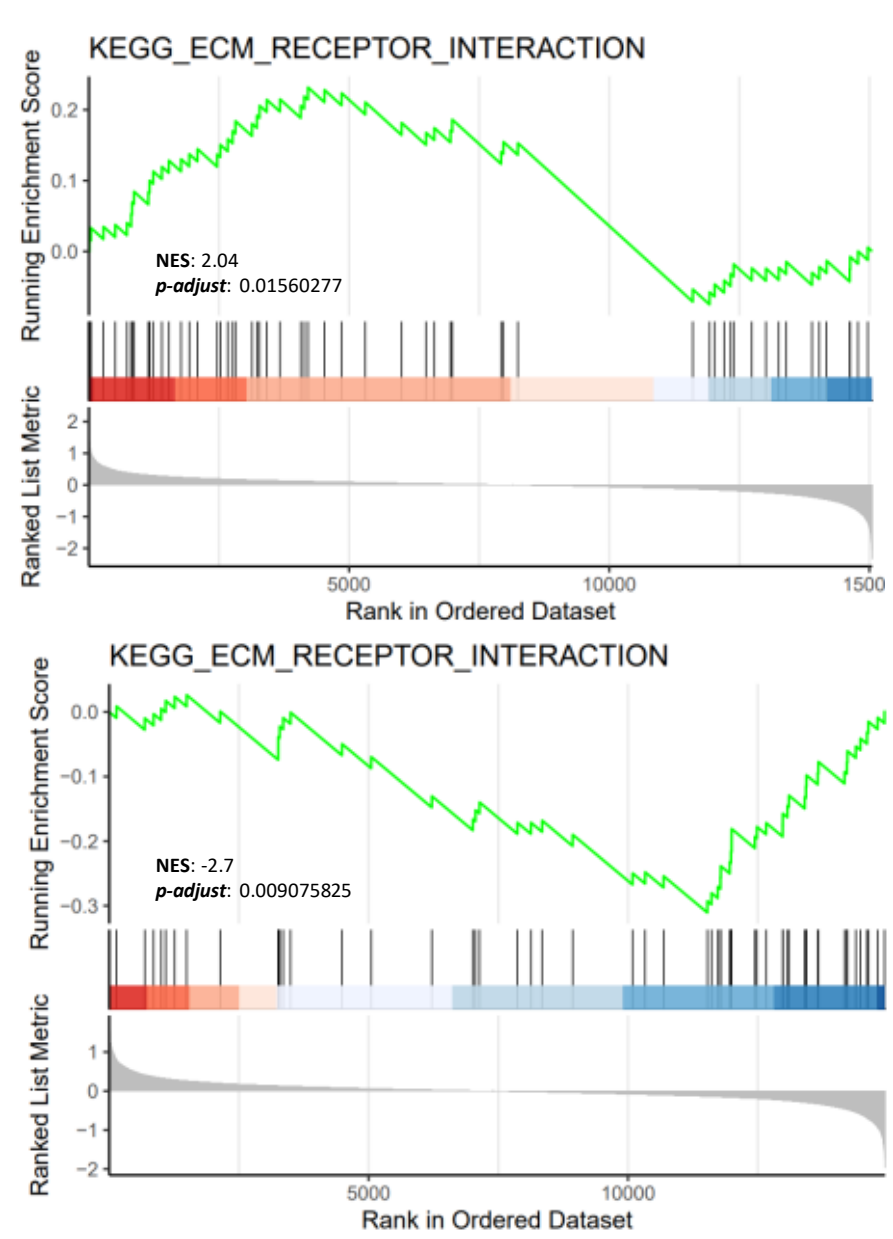

S2E

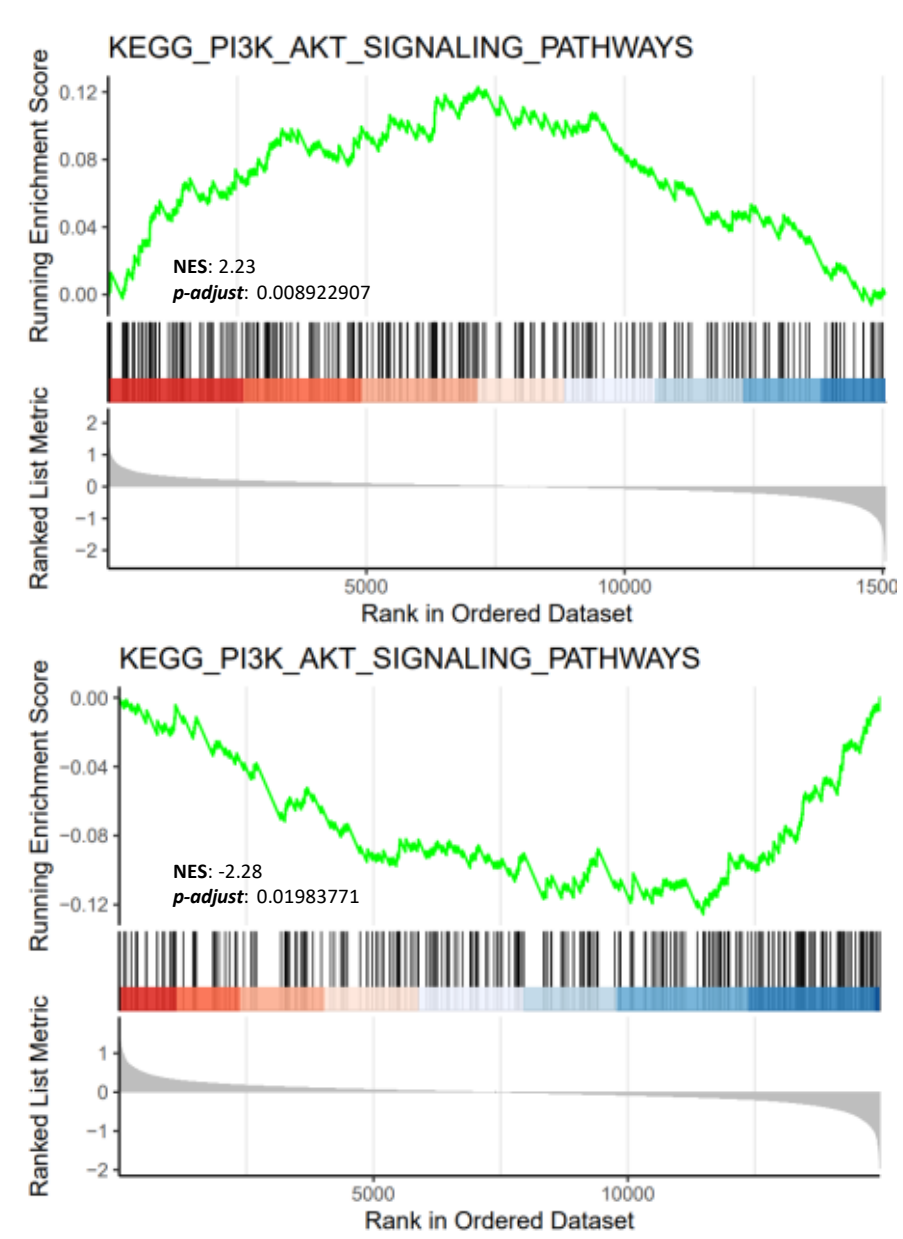

S2F

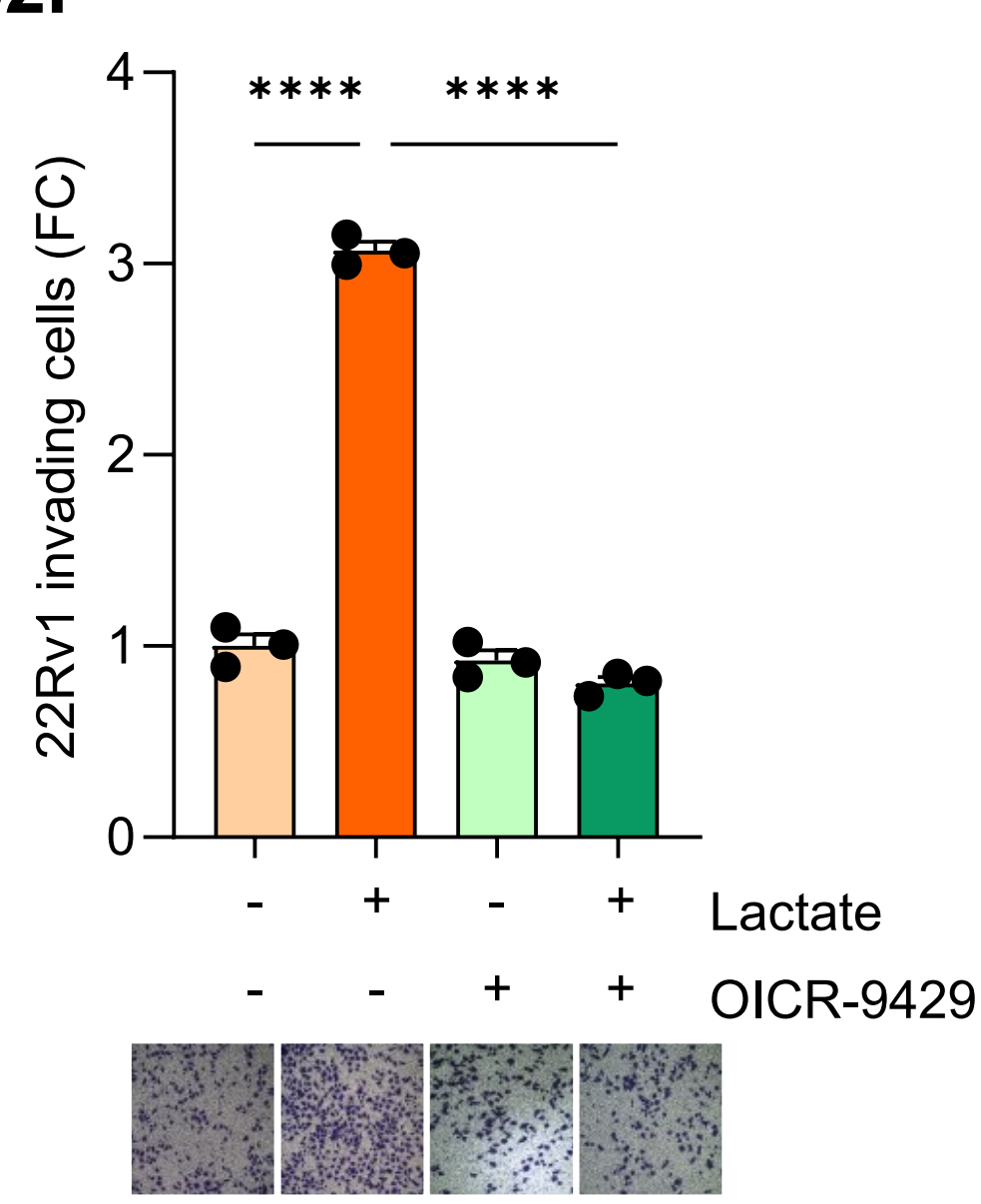

S2G

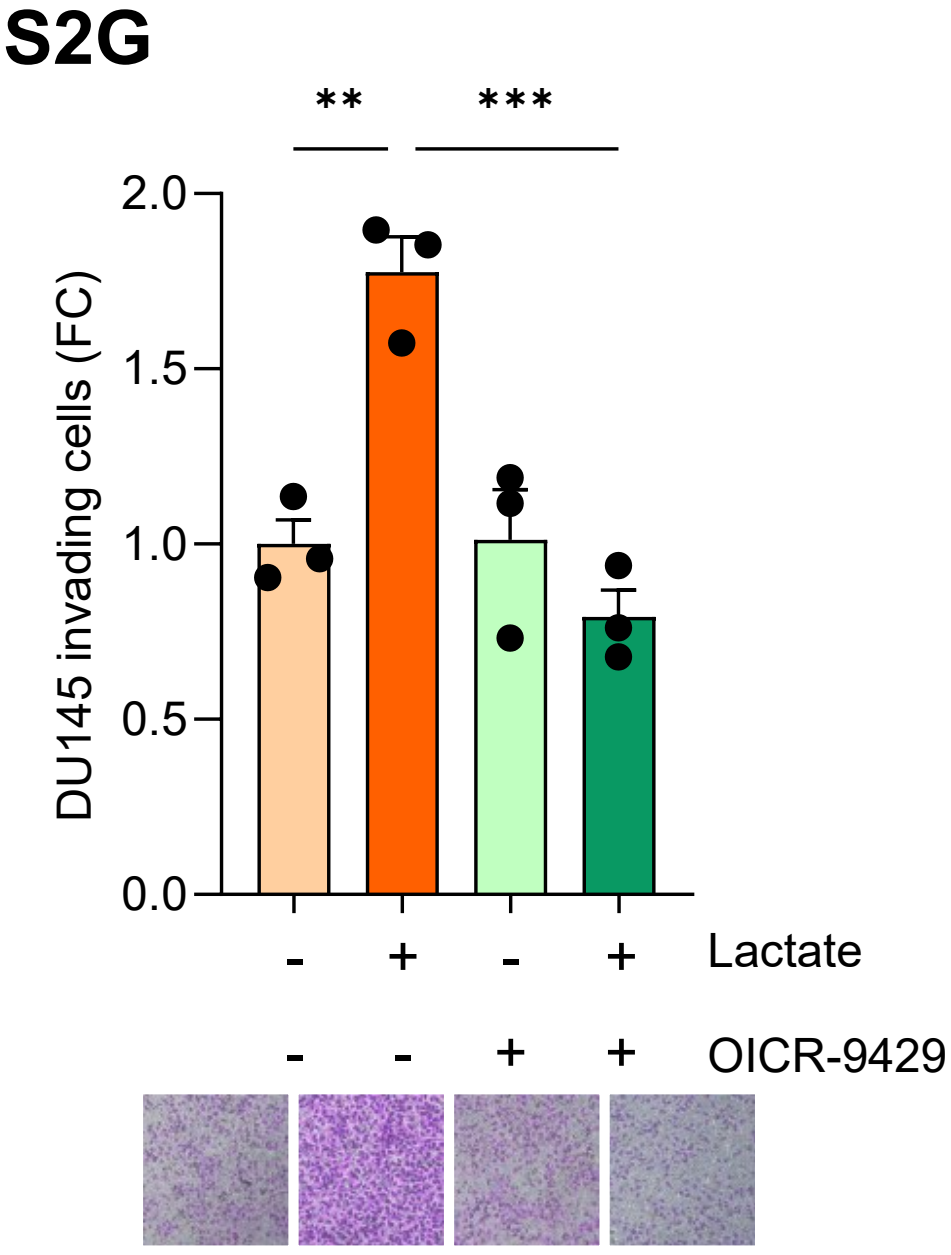

S3A

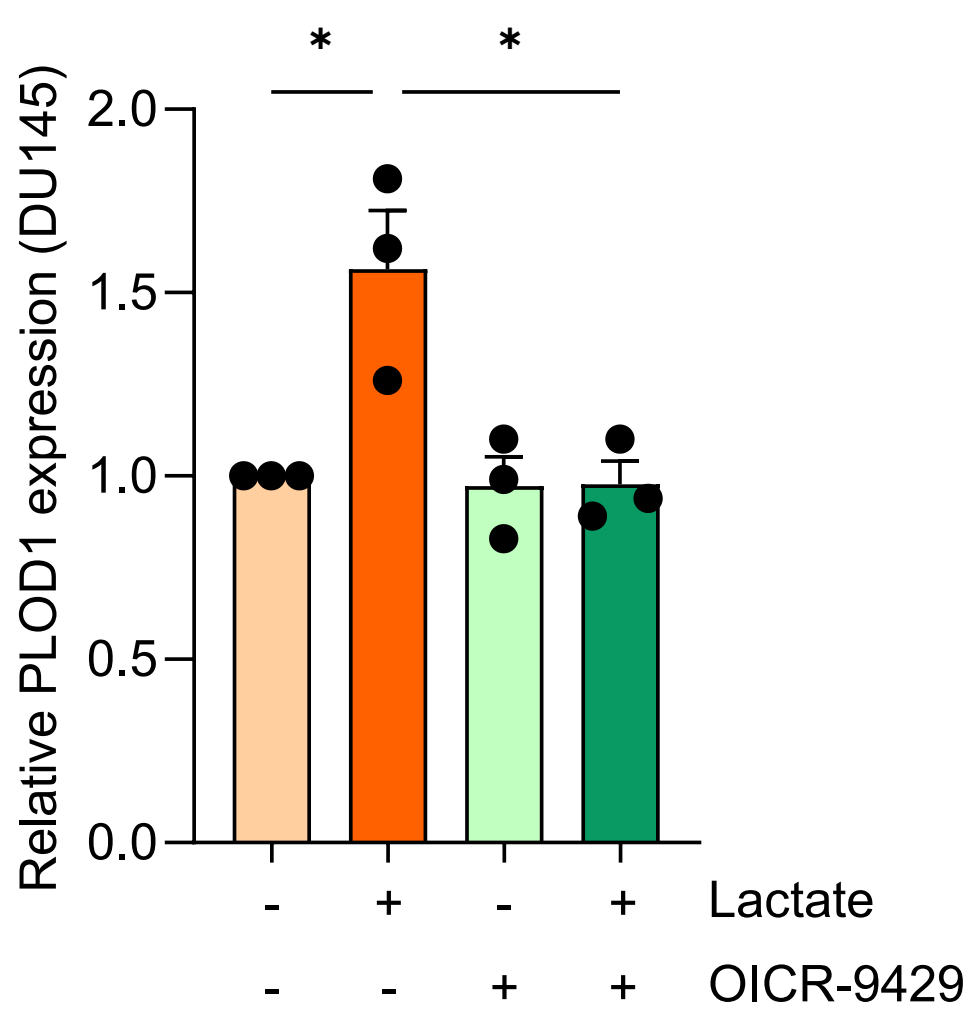

S3B

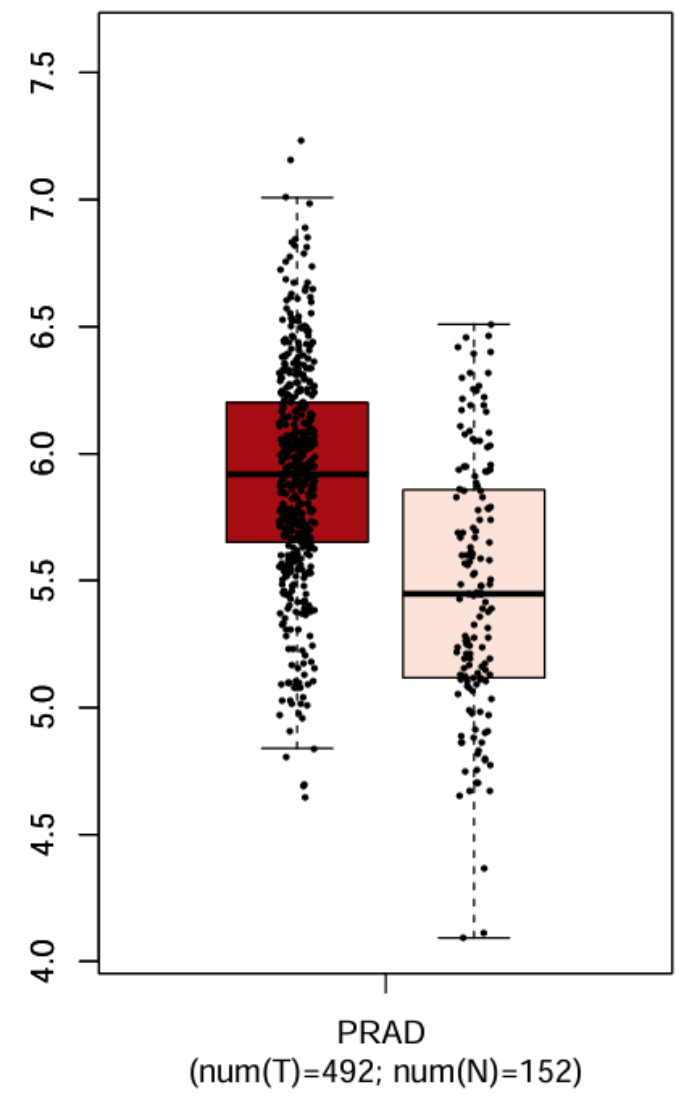

S3C

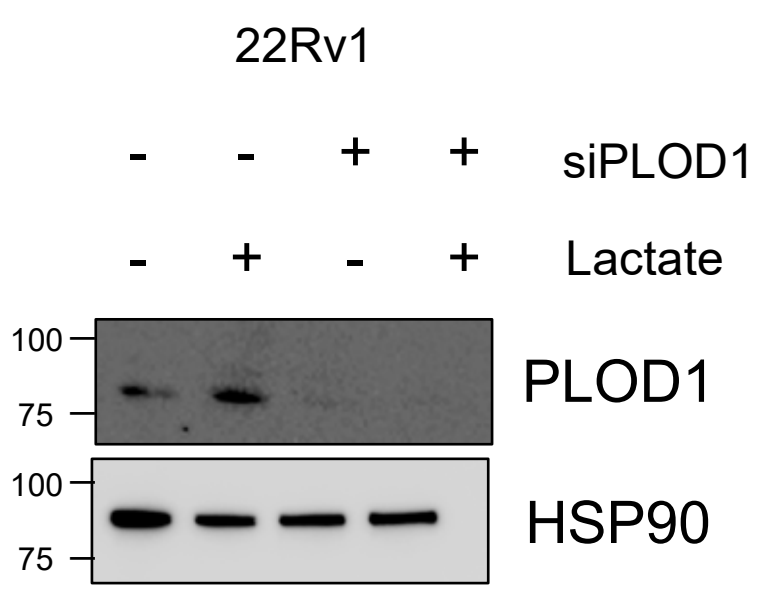

S3D

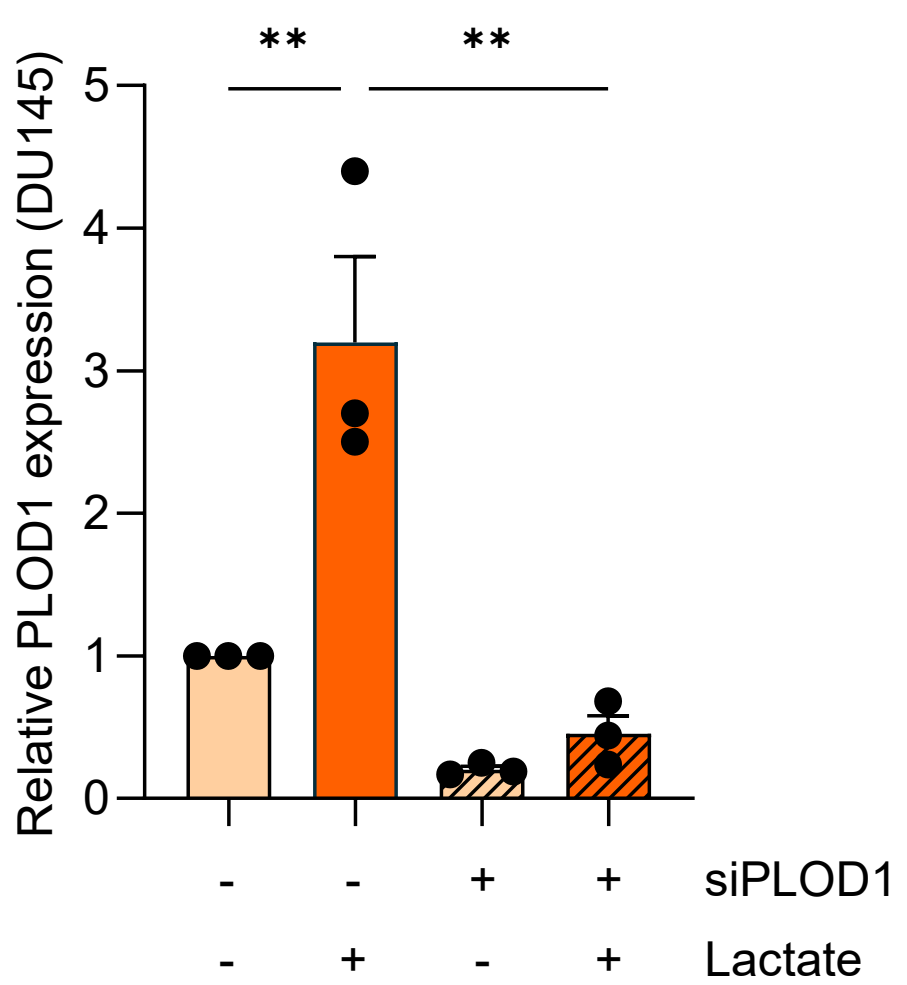

S3E

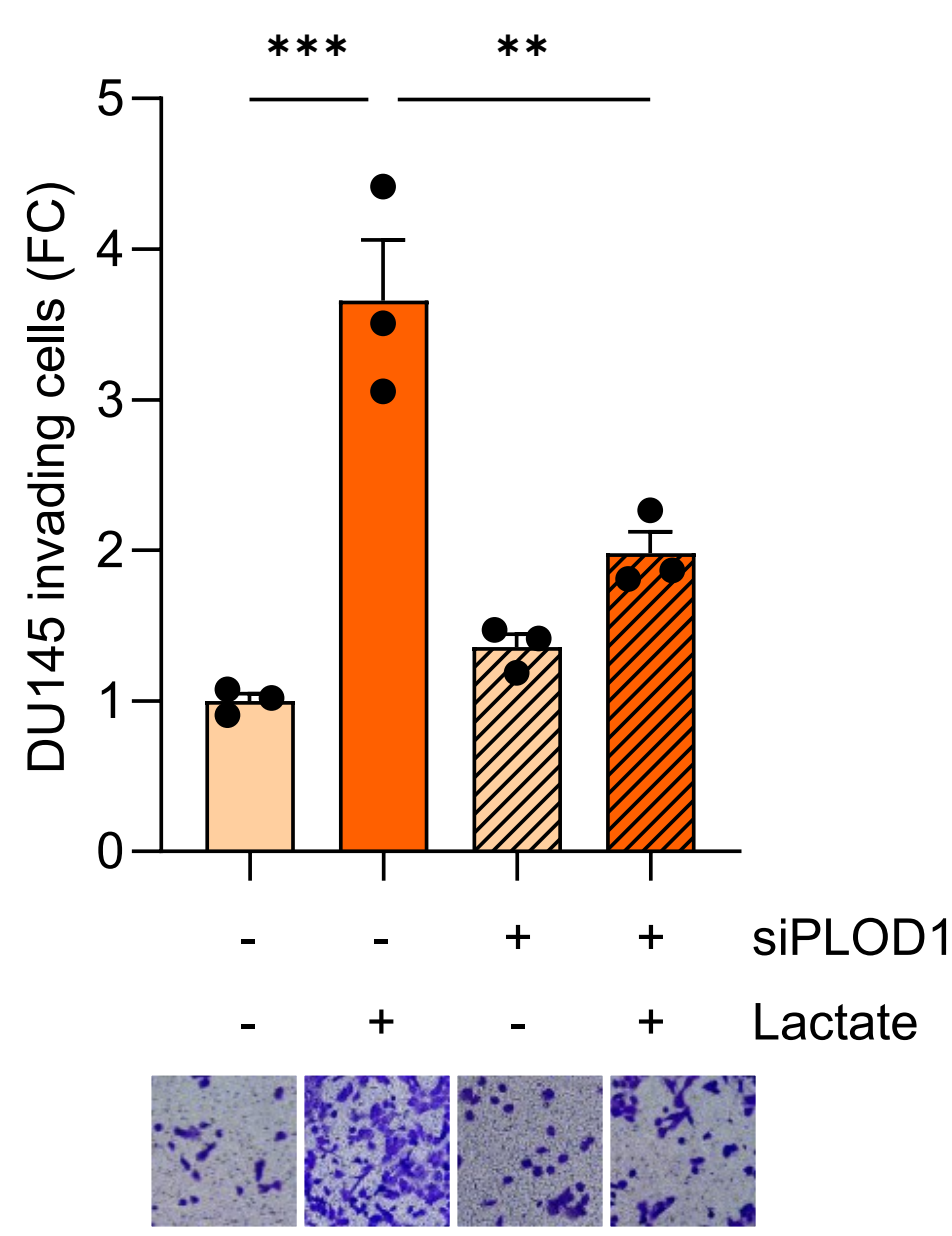

Supplement: Supplementary file 1 [file 41419_2026_9091_MOESM1_ESM.pdf]
