# Supplementary material for: Lactate-induced H3K4me3 histone mark promotes PLOD1-driven prostate cancer invasion
Source: Cell Death Dis. 2026 Jul 7;17(1):843. doi: 10.1038/s41419-026-09091-4 (PMC13620169; doi:10.1038/s41419-026-09091-4)

1A

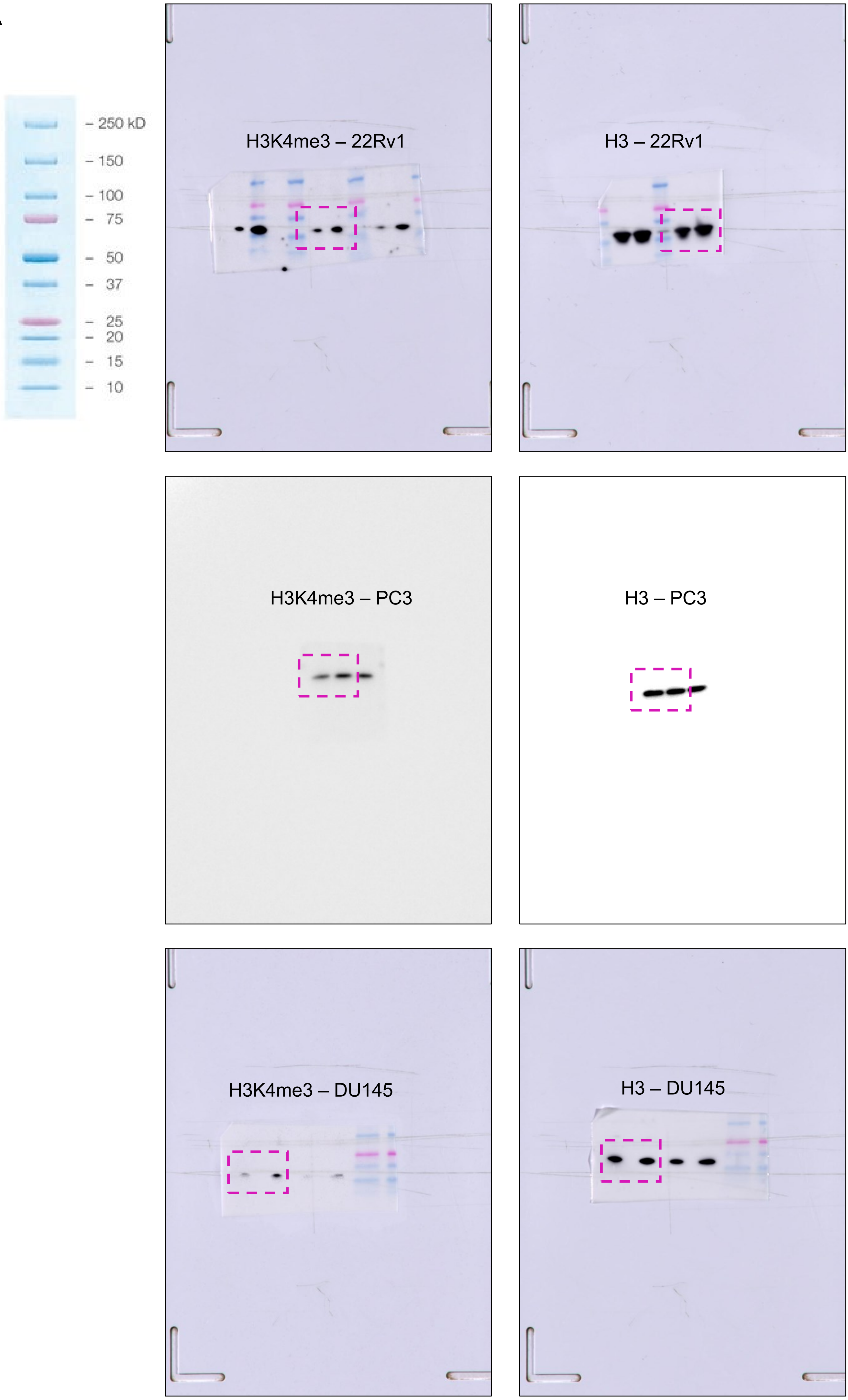

– 250 kD
– 150
– 100
– 75
– 50
– 37
– 25
– 20
– 15
– 10
H3K4me3 – 22Rv1
H3 – 22Rv1
H3K4me3 – PC3
H3 – PC3
H3K4me3 – DU145
H3 – DU145

1A

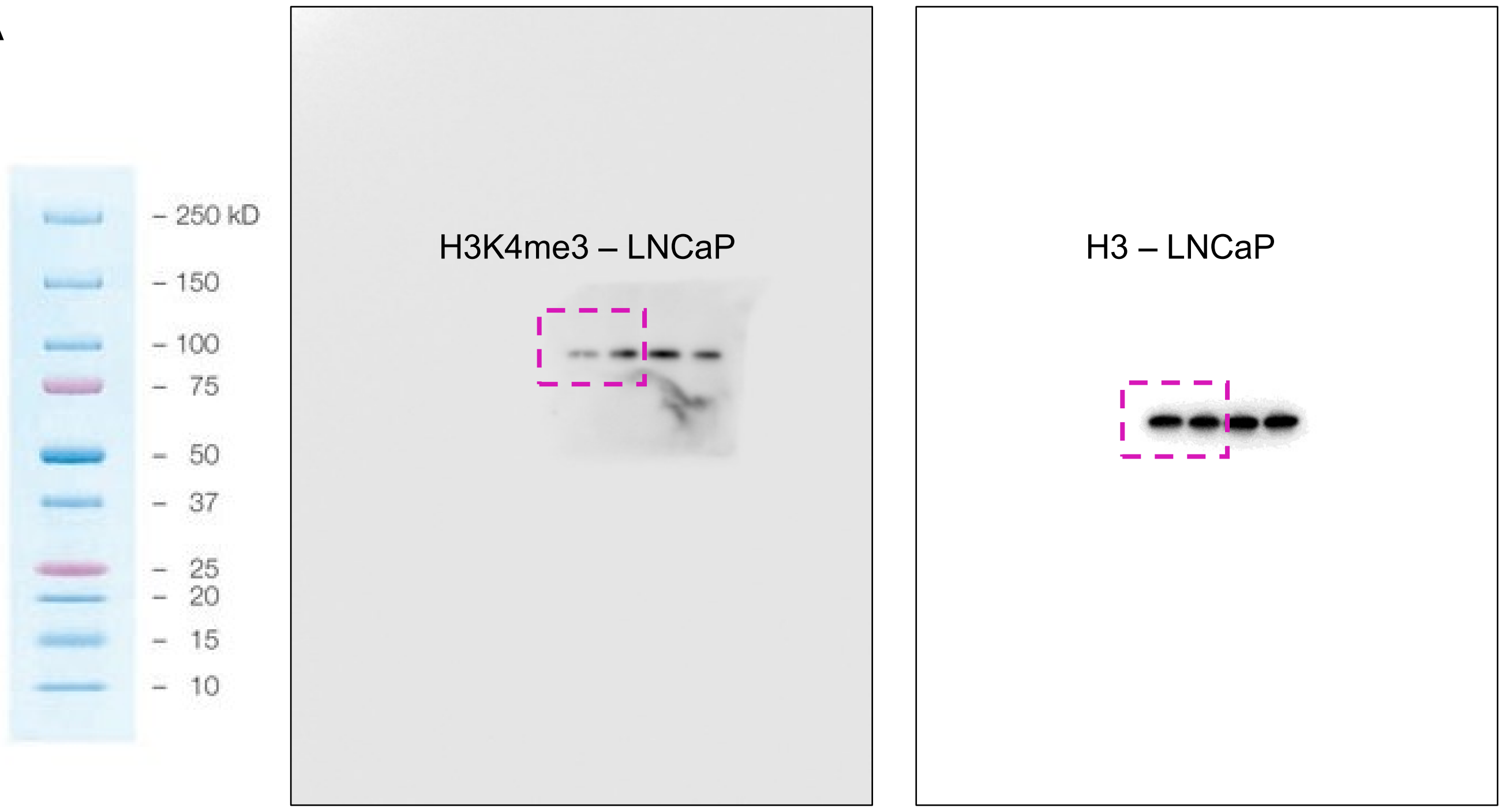

– 250 kD
– 150
– 100
– 75
– 50
– 37
– 25
– 20
– 15
– 10
H3K4me3 – LNCaP
H3 – LNCaP

1B

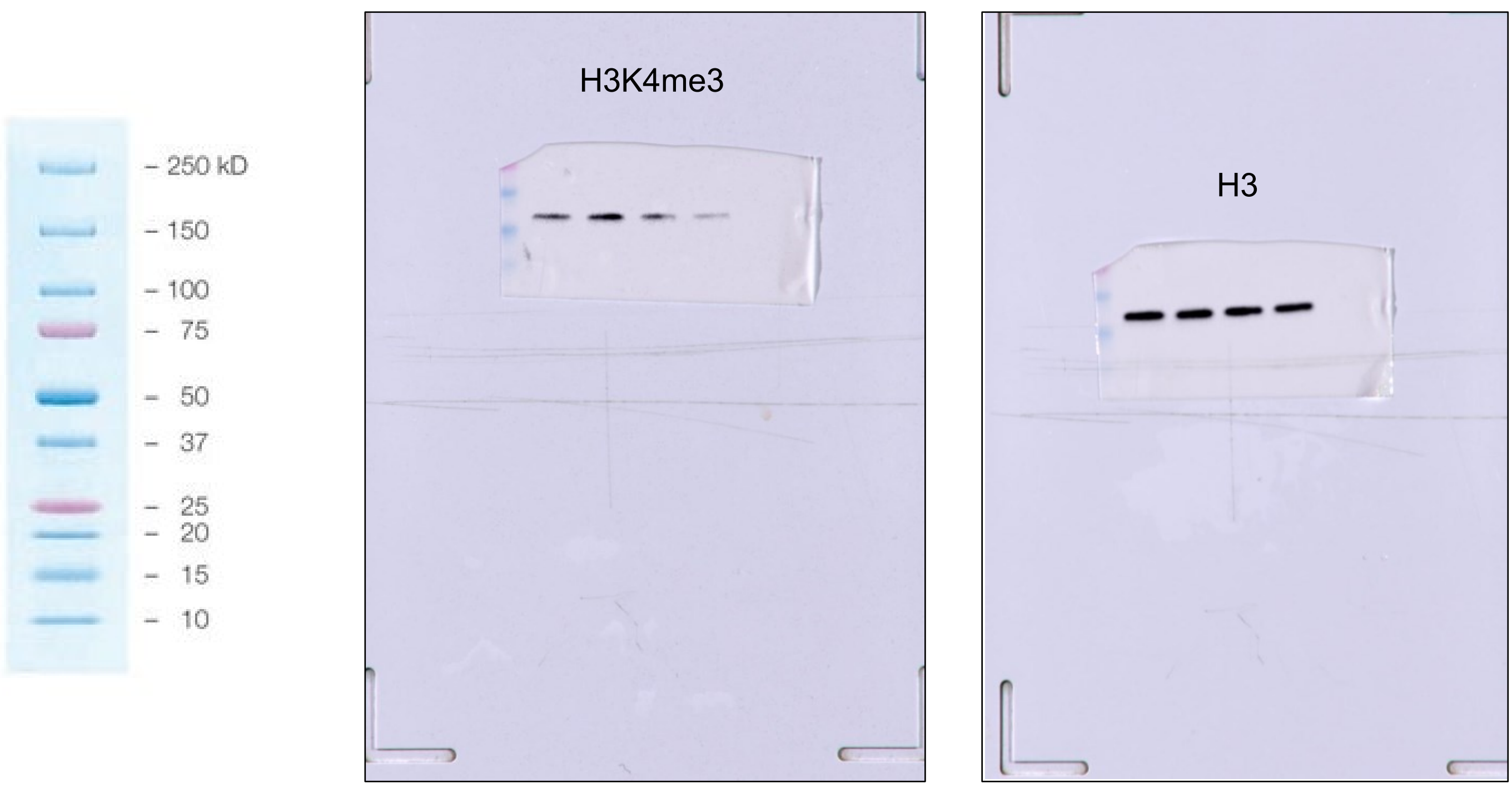

– 250 kD
– 150
– 100
– 75
– 50
– 37
– 25
– 20
– 15
– 10
H3K4me3
H3

2D

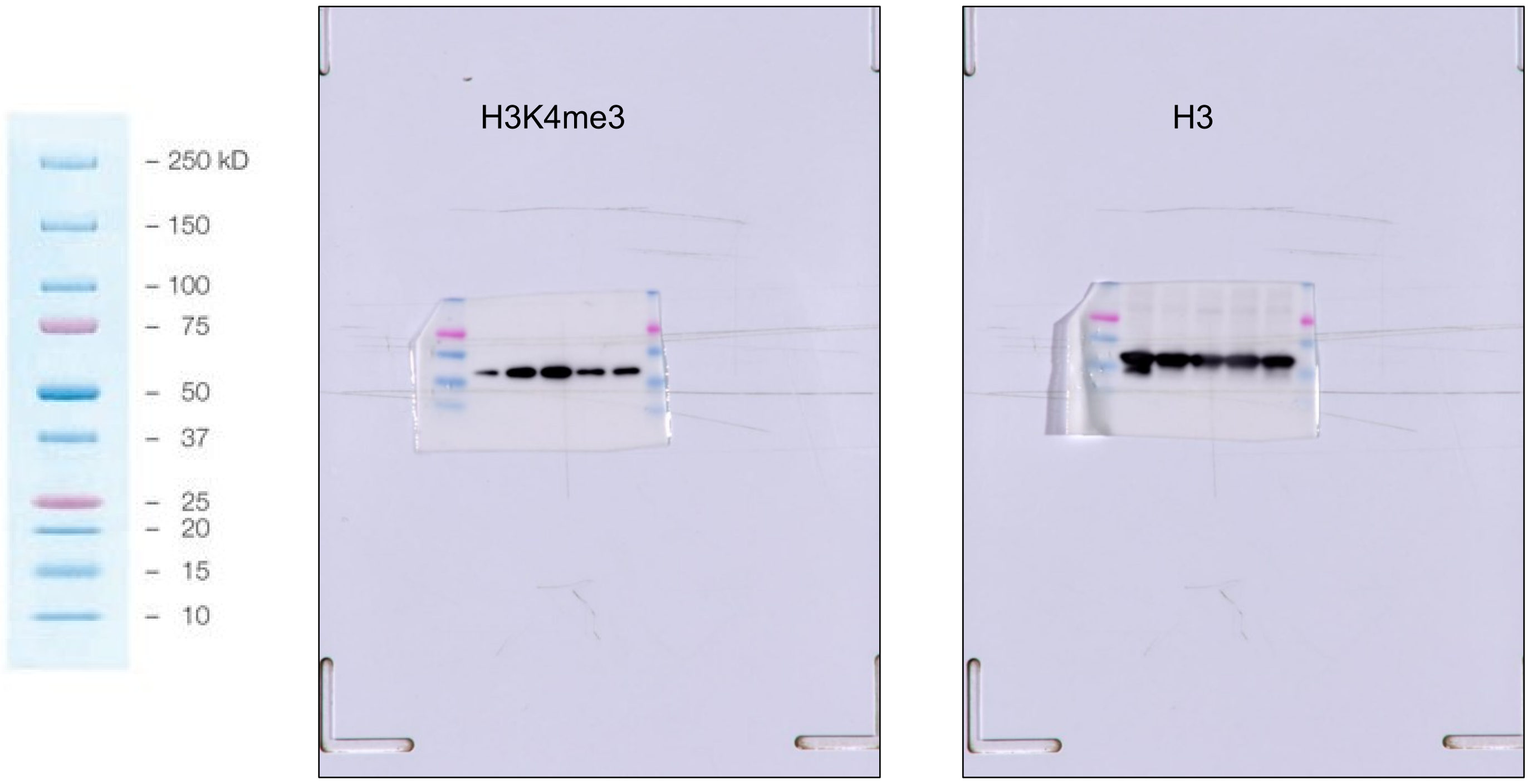

S1A

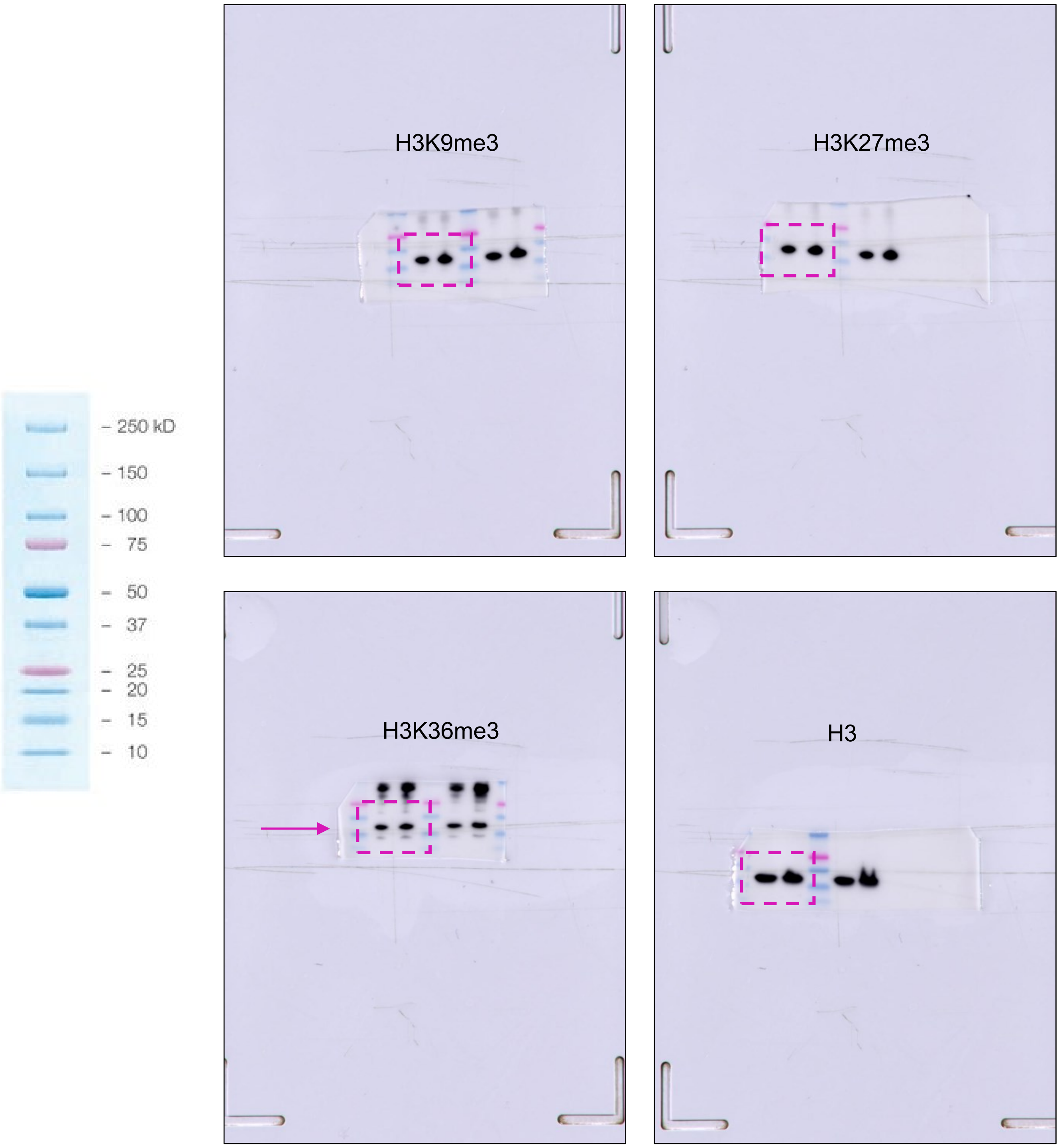
250 kD
150
100
75
50
37
25
20
15
10
H3K9me3
H3K27me3
H3K36me3
H3

S2A

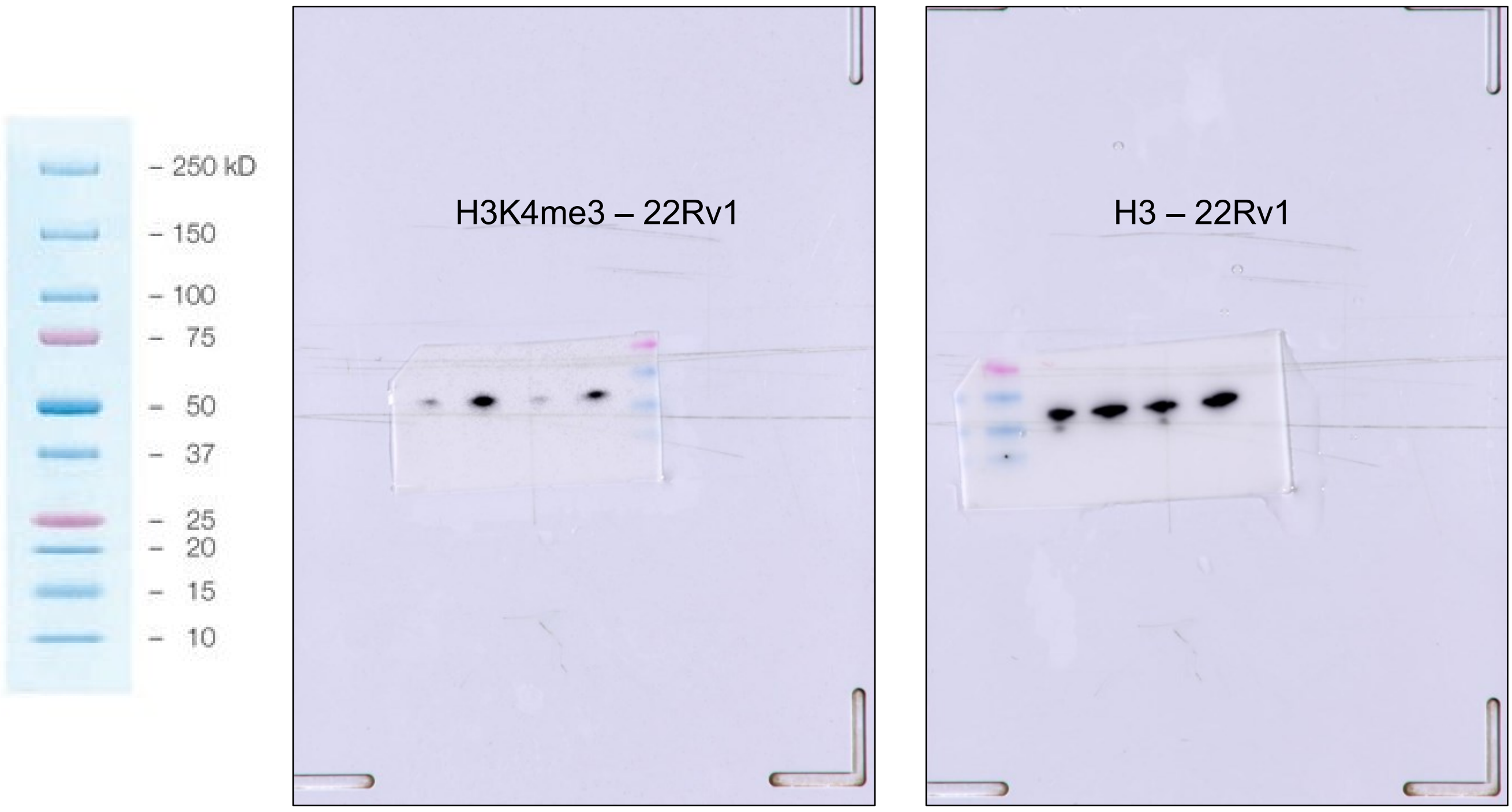
250 kD
150
100
75
50
37
25
20
15
10
H3K4me3 – 22Rv1
H3 – 22Rv1

S2B

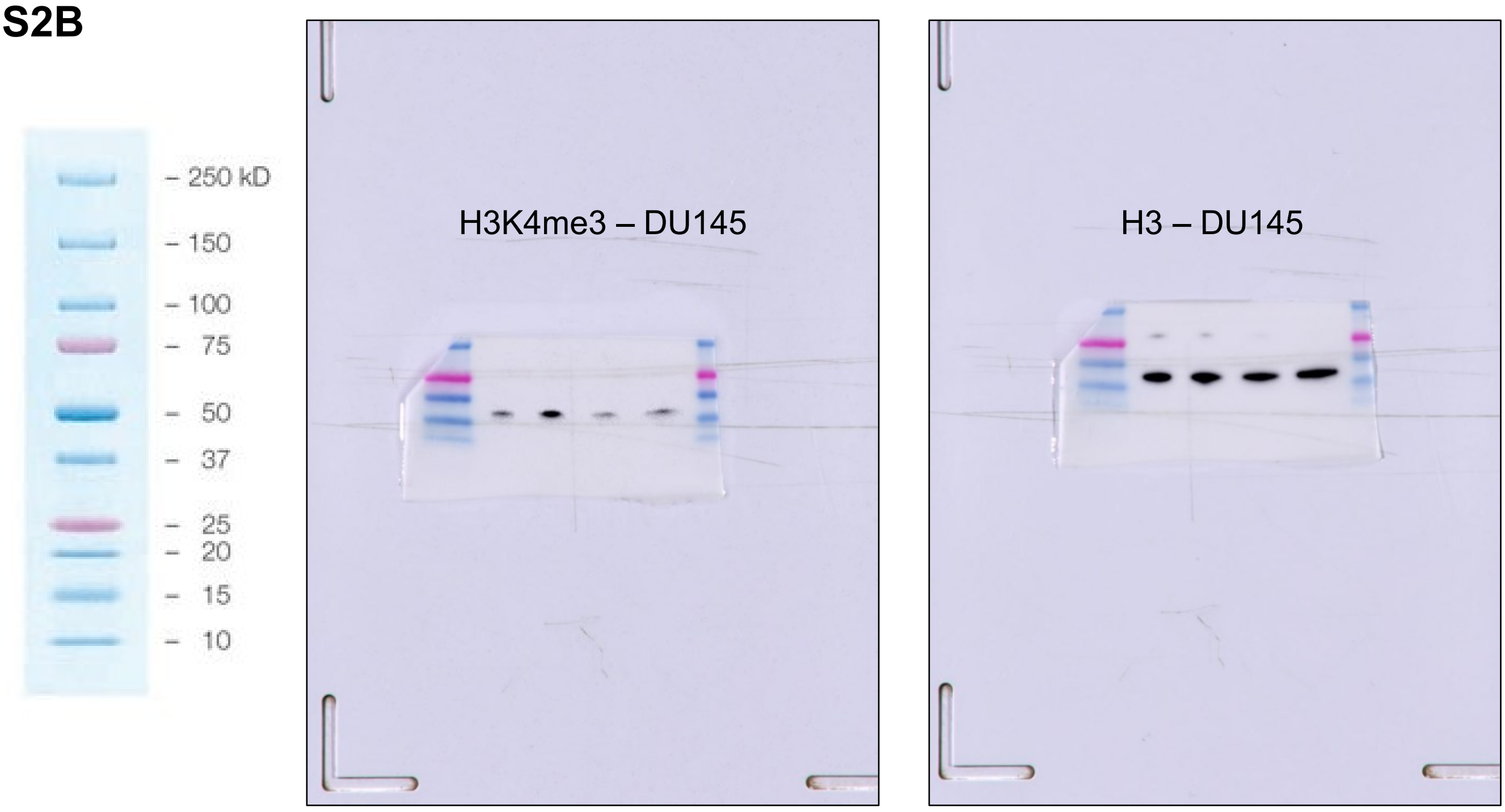
250 kD
150
100
75
50
37
25
20
15
10
H3K4me3 – DU145
H3 – DU145

S3C

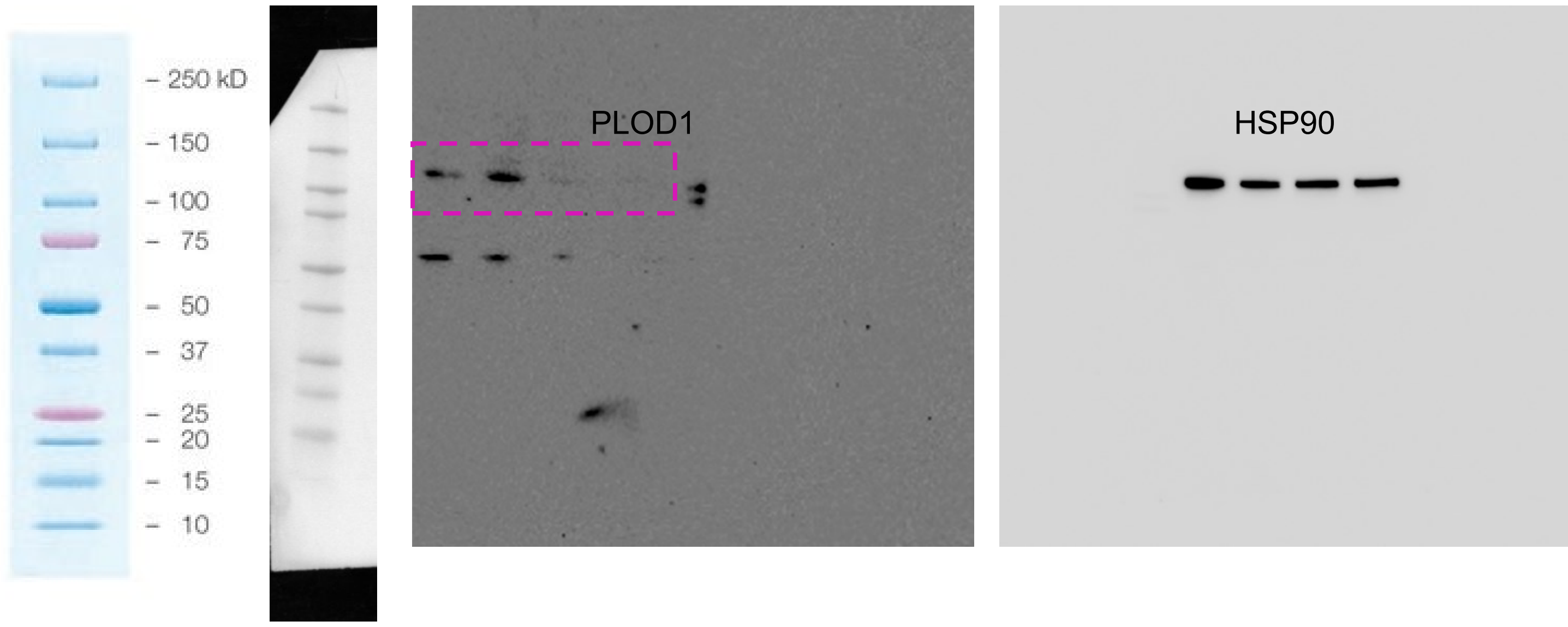

Supplement: Supplementary file 2 [file 41419_2026_9091_MOESM5_ESM.pdf]
