# Supplementary material for: Lactate-induced H3K4me3 histone mark promotes PLOD1-driven prostate cancer invasion
Source: Cell Death Dis. 2026 Jul 7;17(1):843. doi: 10.1038/s41419-026-09091-4 (PMC13620169; doi:10.1038/s41419-026-09091-4)
Supplement: Supplementary file 3 [file 41419_2026_9091_MOESM2_ESM.doc]

**Lactate-induced H3K4me3 histone mark promotes PLOD1-driven prostate cancer invasion**

Marta Iozzo ^1,4^, Luigi Ippolito ^1^, Erica Pranzini ^1^, Simone Romagnoli ^1^, Giulia Gangarossa ^1^, Caterina Grillo ^1^, Sara Roccabianca ^1^, Giada Sandrini ^2^, Matteo Benelli ^1^, Giuseppina Comito ^1^, Elisa Giannoni ^1,3^, Paola Chiarugi ^1,3,4^

*^1^ Department of Experimental and Clinical Biomedical Sciences, University of Florence, Viale Morgagni 50, 50134, Florence, Italy.*

*^2^ Division of Theoretical Systems Biology, German Cancer Research Center (DKFZ), Heidelberg, Germany*

*^3^ These authors contributed equally*

*^4^ Correspondence: paola.chiarugi@unifi.it;* [*marta.iozzo@unifi.it*](mailto:marta.iozzo@unifi.it)

**Supplementary figure and table legends**

**Figure S1. LA selectively induces H3K4me3 and enhances PCa cell invasion *via* metabolic cues.**

(**A**) H3K9me3, H3K27me3 and H3K36me3 levels evaluated by WB analysis in 22Rv1 cells treated with or without LA (20 mM, 48 h). H3 was used as loading control. Densitometric analysis of H3K4me3/H3 ratio is shown (n = 3). (**B**) Invasion assay performed on 22Rv1 cells treated with LA with or without MCT1 inhibitor. Representative pictures are shown, magnification 20x. (**C**) IHC analysis of H3K4me3 expression in a stromalized PCa 3D *in vitro* model. Representative image shown, scale bar: 20 μm. (**D**) Ratios of succinate/α-KG (left) and fumarate/α-KG (right) derived from GC-MS peak area analysis in DU145 cells treated ± LA. (**E**) Invasion assay performed on DU145 cells treated with LA, DM-succinate, or DM-αKG. Representative pictures are shown, magnification 20x.

Data are represented as means ± SEM of n = 3 biological replicates. Statistical significance was assessed using unpaired two-tailed t-test (D) or one-way ANOVA followed by Tukey’s multiple comparisons test (B, E) (*P < 0.05; **P < 0.01; ***P < 0.001; ****P < 0.0001).

**Figure S2. LA-induced H3K4me3 is associated with pro-invasive transcriptional programs and enhanced invasiveness in PCa cells.**

(**A-B**) H3K4me3 protein levels evaluated by western blot analysis in 22Rv1 and (B) DU145 treated with LA with or without OICR-9429 (10 μM). H3 was used as a loading control. Densitometric analysis of H3K4me3/H3 ratio is shown (n = 3). (**C-E**) GSEA plots illustrating the enrichment of the selected pathways under two experimental comparisons: LA *vs* Control (*upper* panel) and LA + OICR-9429 *vs* LA (*lower* panel). Each plot displays the ranked distribution of *Cell Adhesion-, ECM-Receptor Interaction-* and *PI3K-AKT Signaling*-related genes based on differential expression, with enrichment scores reflecting the degree of coordinated upregulation or downregulation of the gene set in the respective conditions (n = 4). (**F-G**) Invasion assay performed on 22Rv1 (F) and (G) DU145 cells treated with LA ± OICR-9429. Representative pictures are shown, magnification 20x.

Data are represented as means ± SEM of n = 3 biological replicates unless otherwise stated. Statistical significance was determined by p adjusted by FDR (C-E) or one-way ANOVA followed by Tukey’s test (F-G) (*P < 0.05; **P < 0.01; ***P < 0.001; ****P < 0.0001).

**Figure S3. PLOD1 is a downstream effector of the LA-H3K4me3 axis and contributes to invasiveness in DU145 cells.**

(**A**) qRT-PCR analysis of *PLOD1* mRNA levels in DU145 cells treated with LA ± OICR-9429. (**B**) Box plot analysis of *PLOD1* expression in normal *vs* PCa tumors using *GEPIA2* (TCGA PRAD dataset). Each dot corresponds to an individual specimen (Tumor = 492, Normal = 152). (**C**) PLOD1 protein levels evaluated by western blot analysis in 22Rv1 silencing or not with siPLOD1 (20 nM). HSP90 was used as a loading control. (**D**) qRT-PCR analysis of *PLOD1* mRNA levels in DU145 PLOD1-silenced cells and treated ± LA. (**E**) Invasion assay performed on DU145 cells treated with LA ± PLOD1 silencing. Representative pictures are shown, magnification 20x.

Data are represented as means ± SEM of n = 3 biological replicates. Statistical significance was determined by one-way ANOVA followed by Tukey’s test (A, D-E) (*P < 0.05; **P < 0.01; ***P < 0.001).

**Supplementary table 1.** DEGs between LA-treated cells and control conditions using the DESeq2 package.

**Supplementary table 2.** Representative genes of Cluster E.
